# Catalogue of stage-specific transcripts in *Ixodes ricinus* and their potential functions during the tick life-cycle

**DOI:** 10.1186/s13071-020-04173-4

**Published:** 2020-06-16

**Authors:** Pavlina Vechtova, Zoltan Fussy, Radim Cegan, Jan Sterba, Jan Erhart, Vladimir Benes, Libor Grubhoffer

**Affiliations:** 1 Faculty of Science, University of South Bohemia, České Budějovice, Czech Republic; 2 Biology Centre CAS, Institute of Parasitology, České Budějovice, Czech Republic; 3 BIOCEV, Faculty of Science, Charles University, Prague, Czech Republic; 4 Department of Plant Developmental Genetics, Institute of Biophysics of the Czech Academy of Sciences, Brno, Czech Republic; 5 GeneCore, EMBL, Heidelberg, Germany

**Keywords:** *Ixodes ricinus*, Tick development, Transcriptome assembly, Reference gene validation, Life stage

## Abstract

**Background:**

The castor bean tick *Ixodes ricinus* is an important vector of several clinically important diseases, whose prevalence increases with accelerating global climate changes. Characterization of a tick life-cycle is thus of great importance. However, researchers mainly focus on specific organs of fed life stages, while early development of this tick species is largely neglected.

**Methods:**

In an attempt to better understand the life-cycle of this widespread arthropod parasite, we sequenced the transcriptomes of four life stages (egg, larva, nymph and adult female), including unfed and partially blood-fed individuals. To enable a more reliable identification of transcripts and their comparison in all five transcriptome libraries, we validated an improved-fit set of five *I. ricinus*-specific reference genes for internal standard normalization of our transcriptomes. Then, we mapped biological functions to transcripts identified in different life stages (clusters) to elucidate life stage-specific processes. Finally, we drew conclusions from the functional enrichment of these clusters specifically assigned to each transcriptome, also in the context of recently published transcriptomic studies in ticks.

**Results:**

We found that reproduction-related transcripts are present in both fed nymphs and fed females, underlining the poorly documented importance of ovaries as moulting regulators in ticks. Additionally, we identified transposase transcripts in tick eggs suggesting elevated transposition during embryogenesis, co-activated with factors driving developmental regulation of gene expression. Our findings also highlight the importance of the regulation of energetic metabolism in tick eggs during embryonic development and glutamate metabolism in nymphs.

**Conclusions:**

Our study presents novel insights into stage-specific transcriptomes of *I. ricinus* and extends the current knowledge of this medically important pathogen, especially in the early phases of its development.
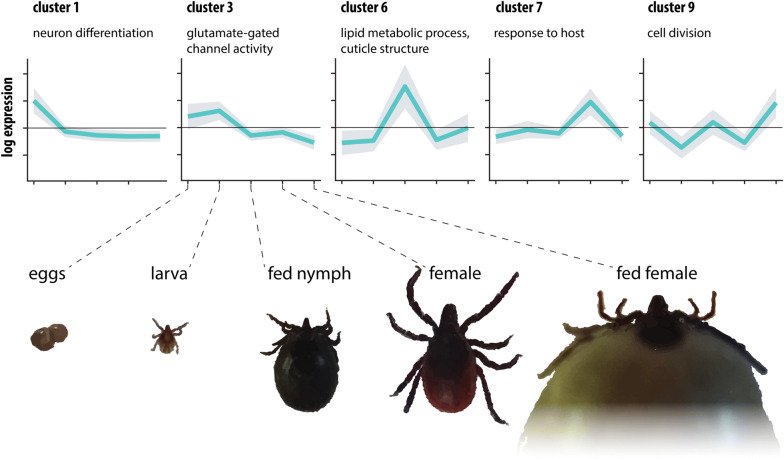

## Background

The European tick *Ixodes ricinus* is a common blood-feeding arthropod transmitting several widespread human pathogens, including the spirochaete *Borrelia burgdorferi* causing Lyme disease, tick-borne encephalitis virus (TBEV), the causative agent of human encephalitis and meningitis, *Anaplasma phagocytophilum*, an intracellular alpha-proteobacterium causing granulocytic anaplasmosis, and *Rickettsia* spp. causing spotted fever syndrome [1–4]⁠. As the natural distribution and activity of *I. ricinus* have been augmenting over past decades, so have the emergence and manifestation of tick-borne diseases. Worldwide, there are about 10,000 cases of tick-borne encephalitis [5] and 85,000 cases of Lyme disease [6]⁠, reported annually and these epidemiological numbers have been raising attention with respect to public health, economy or tourism [7–11]⁠.⁠ The life-cycle of arthropod-borne pathogens is tightly bound to the life-cycle of their vectors/hosts and thus understanding the life-cycle of a vector organism often reveals important aspects of vector-pathogen dynamics including the factors influencing disease transmission to final hosts.

Unlike other haematophagous arthropods, such as mosquitoes or flies, ticks exhibit a complex and rather long life-cycle and they usually require feeding on several host organisms for its completion. *Ixodes* ticks are able to complete their life-cycle within 3–6 years in wildlife depending on environmental conditions [12]. During its development, the tick hatches from an egg and undergoes metamorphosis and moults to the next active life stages: larva, nymph, or adult [13, 14]. Each moulting is preceded by blood-feeding on the respective host; the selection of host species is perhaps the broadest of all ticks ranging from small mammals, birds, and reptiles in immature stages to large mammals in adult ticks [15]. The time intervals for the completion of each life stage vary and are greatly influenced by many factors such as season, host abundance, selection of host species, or climatic conditions [16]. The feeding itself lasts 3–5 days in a larval stage, 4–7 days in nymphs, and 7–11 days in adult females [17]. Adult females mate with adult males during feeding on the host to accomplish reproduction. A laid egg batch contains, on average, 2000–2500 eggs [18]⁠ but the number of eggs in one batch can reach up to 4000 [19]. The reason for an extraordinarily long life-cycle of *I. ricinus* is arguably its use of three hosts, in particular when the tick drops off the host after each blood meal and undergoes metamorphosis and moulting off the host [14]⁠. Furthermore, the absence of a host or suboptimal microclimatic conditions (e.g. low temperature) drive the tick to enter a diapause, which can be induced in any life stage and contributes to the extension of its life-cycle [20].

Longevity along with the blood-feeding ectoparasitic life strategy of ticks must have been preceded by many adaptations, differing from those of blood-feeding insects and including features in the regulation of development and metamorphosis that are yet a matter of debate [13].

The efforts to describe factors controlling the development of other arthropod vectors, such as vectors of malaria (*Anopheles gambiae*) and yellow fever (*Aedes aegypti*), are mainly focused on the blood-feeding and reveal an upregulation of genes associated with blood-meal processing, peritrophic matrix formation, egg development, and immunity on the organismal level [21, 22]⁠ or in salivary glands [23–26], the latter being regarded as a crucial mediator of pathogen transmission to the mammalian host. Several transcriptomic studies were focusing on molecules that might directly influence feeding, such as haem utilization in ticks [27]⁠ or arthropod proteases, both being essential factors enabling a haematophagous life strategy [28]. Transcriptional regulation of the entire life-cycle, controlling tick ontogenesis and development has not been fully covered to date and existing research has only focused on a specific organ [29]⁠ or a life stage [30].

Due to an increase in its epidemiological importance, *I. ricinus* has become a species featured in many recent transcriptomic studies, the majority of them focused on the transcription in salivary glands and/or midgut, which are the key organs in the tick-borne pathogens’ life-cycle. Studying these organs in response to blood-feeding can be instrumental for the identification of factors that underlie survival and dissemination of pathogens within their vector and their transmission into the final host [24, 31–37]⁠. More specifically, expression analyses using tick haemocytes, the main actors in tick immunity, can unveil the character of the immunity barrier for tick-borne pathogens [29]. Organism-level transcriptomes of feeding stages, on the other hand, can provide a picture of global changes induced by a blood meal [30]⁠, hence a more thorough description of factors driving tick developmental processes throughout its life-cycle will be instrumental in understanding the process of host-seeking and blood-feeding as an integral event in tick development.

In this study, we focused on transcripts associated with development, aiming at the presentation of significant new data of the main processes linked to specific life-stages of *I. ricinus* and functions that are stably expressed. We present a catalogue of transcripts in transcriptome assemblies of several life stages of *I. ricinus* to provide an outline of transcription important for specific time points of tick development and functions in particular life stages. Our data identified transcripts involved in tick embryonic development thereby providing a source of information for research in tick cell lines, a tick model for *in vitro* research derived from tick embryonic cells [38]. This represents a significant contribution, which facilitates an initiation and development of methods largely applicable by means of *in vitro* models such as double-stranded RNA post-transcriptional gene silencing (RNAi) or targeted genome editing using CRISPR/Cas9.

## Methods

### Sample preparation and next-generation sequencing

Both partially fed and unfed life stages of *I. ricinus* were collected in the tick rearing facility of the Institute of Parasitology, Biology Centre CAS, České Budějovice, Czech Republic; partially fed life stages were fed on laboratory guinea pigs obtained at the animal rearing facility therein. The partially fed nymphs were feeding 3–4 days and partially fed females 5–6 days. Under rearing facility conditions, fed females start laying eggs 4 weeks after feeding and the process takes approximately 2 weeks. The eggs were collected immediately after laying and thus represent an early stage of embryogenesis. Larvae were collected after complete hatching of an egg clutch, which usually takes 2–4 weeks. Unfed females were hatching 7–9 weeks after the full engorgement of feeding nymphs. Females were collected after all females had moulted from a batch of nymphs that were feeding simultaneously on laboratory animals. Fed stages were not dissected to remove host blood as haemolymph containing haemocytes and possibly other cells would be lost in the process.

Total RNA was isolated from 3 halves of egg clutches (3 × ~ 600 eggs), 3 batches of larvae hatched from 3 halves of egg clutches (3 × ~600 individuals), partially fed nymphs (3 × 10 individuals), adult (unfed) females (3 × 7 individuals) and partially fed females (3 × 3 individuals) of *I. ricinus* ticks using NucleoSpin RNA II (Macherey-Nagel, Duren, Germany) according to the manufacturer’s instructions. The concentration of RNA was measured using an Implen NanoPhotometer (Implen, Munchen, Germany) and the quality of RNA was determined using a 2100 Bioanalyzer with RNA 6000 Nano kit (Agilent Technologies, Santa Clara, CA, USA). The 3 RNA samples of each life stage were pooled to obtain a single RNA sample per life stage. Each sample is a mixture of three different cohorts of ticks collected in a tick rearing facility in order to cover the genetic variability existing between individuals and to reconstruct the maximum number of transcripts that can be possibly expressed in each life stage. cDNA synthesis and library preparation was carried out using TruSeq DNA Sample Prep kit v2 (PE50 reads) (Illumina, San Diego, CA, USA), followed by sequencing on the HiSeq 2000 platform; both the library preparation and sequencing were performed by the GeneCore facility of the European Molecular Biology Laboratory (EMBL), Heidelberg, Germany.

### Transcriptome assembly and annotation

The PE50 raw reads of all five stages were trimmed off sequencing adapters, short and low-quality reads using Trimmomatic (LEADING:3 TRAILING:3 SLIDINGWINDOW:4:15 MINLEN:36 trimmomatic parameters) [39]. Trimmed reads of all five libraries were used to build a single assembly using Trinity assembler v2.1.1 [40] in order to obtain a highly comprehensive catalogue of complete and high-quality transcripts. Assembly completeness was assessed by mapping the raw reads to the assembly using Bowtie2 v2.3.0 [40]⁠ and Samtools flagstat v1.6 [41], followed by BUSCO v3.0.2 search [42]⁠ using the catalogue of conserved Arthropoda orthologues in protein mode (database last accessed on 7 July 2017). Contigs showing high similarity to taxonomic groups other than Panarthropoda according to the BlobTools v1.0 pipeline [43] were omitted as possible contaminants.

The contigs of transcriptome assembly were conceptually translated into protein sequences in all six frames. To retrieve annotations, contigs were queried in protein space against TrEMBL, Swiss-Prot, and non-redundant protein database (nr) (downloaded on 30 July 2017) using blastp of BLAST v2.6.0+ with default parameters except for the e-value set to 1 × 10^−4^, returning a maximum of 10 best hits (-max_target_seqs 10). Using a custom Python3 script, the data were integrated with the annotations retrieved by InterProScan v5.36-75.0 [44]⁠, which also provided the gene ontology (GO) terms for contigs. Some annotations and GO terms were transferred from the *I. ricinus* proteome at UniProt, with the criterion for an assignment being > 90% sequence identity on the protein level.

### Validation of assembly completeness

Assembly completeness and comprehensiveness was assessed by the search of transcripts showing tissue-specific expression. The query protein sequences were downloaded from GenBank (database last accessed on 11 February 2020) (see Table 1 for accession numbers). Blastp of BLAST v2.6.0+ with default parameters was used to search a transcriptome assembly translated into protein sequences in all six frames. The best BLAST hits were collected and their corresponding nucleotide sequences were retrieved from *I. ricinus* transcriptome assembly. Alignments of query sequences and their best BLAST hits were constructed using MAFFT 1.4.0 [45]⁠ integrated within Geneious Prime 2020.0.5 (https://www.geneious.com).

**Table 1:** Genes used for the validation of assembly completeness. The tissue specificity and supporting references of the listed genes in *I. ricinus* are also provided

Gene	Accession no. of nucleotide/protein query sequence	Tissue	Reference
IrCD 1	EF428204.1/ABO26561.1	Midgut	[54]
IrCD 2	HQ615697.1/ADU03674.1	Midgut	[54]
IrCD 3	HQ615698.1/ADU03675.1	Midgut	[54]
Iris	AJ269658.2/CAB55818.2	Salivary glands	[55]
Ixoderin A	AY341424.1/AAQ93650.1	Haemocytes, salivary glands, midgut	[56]
Ixoderin B	AY643518.3/AAV41827.2	Salivary glands	[56]

### Reference genes validation

Three individual batches (biological replicates) of eggs (3 × ~ 600 individuals), larvae (3 × ~ 600 individuals), nymphs (3 × 15 individuals), partially fed nymphs (3 × 10 individuals), females (3 × 7 individuals), and partially fed females (3 × 3 individuals) of *I. ricinus* were collected in the tick rearing facility as above. Partially fed stages were dissected and host blood washed off in Ringer physiological solution according to Glaser [46]⁠. Removal of host blood was important as it can inhibit the PCR reaction; since the expression of reference genes is expected to be at similar levels in all tissues, the absence of haemolymph, in this case, did not affect the results. Ticks were homogenized in a mixer mill MM 400 (Retsch, Haan, Germany) with steel beads in LBS buffer and RNA was isolated using NucleoSpin RNA Plus (Macherey-Nagel) according to the manufacturer’s instructions. The concentration of RNA was measured using an Implen NanoPhotometer (Implen). cDNA was synthesized using ProtoScript II First Strand cDNA Synthesis Kit (New England Biolabs, Ipswich, MA, USA). Primers for reference genes were designed according to *I. ricinus* transcripts showing the highest sequence identity with publicly available sequences of *I. scapularis* (see Additional file 1: Table S1 for primer sequences) as determined by blastn of BLAST v2.6.0+ with default parameters. qPCR reactions for the reference gene expression assay in each life stage were prepared using the qPCR 2× SYBR Master Mix (Top-Bio, Vestec, Czech Republic) with 20 ng of total RNA transcribed to cDNA as an input for each reaction. The qRT-PCR reaction and fluorescence acquisition were performed using the LightCycler 480 Real-Time PCR System cycler (Roche, Basel, Switzerland) and the resulting Cq values were recorded and retrieved using the LightCycler 480 Software (release 1.5.0 SP4; Roche, Mannheim, Germany). The validation of reference genes was performed using the BestKeeper v.1 Microsoft Excel-based tool [47]⁠. Gene stability ranking is based on the calculation of pairwise variation of candidate reference genes among samples of different life stages and their biological replications as the standard deviation of log_2_-transformed expression ratios [48]⁠.

### The comparison of transcriptomes of *Ixodes ricinus* life stages

The reads of the common assembly were redistributed among the tick life stages and their read counts were assigned to the transcripts of the individual life stages in order to facilitate identification and annotation of the transcripts and to enable enrichment analysis and visualization. Transcript quantification was not applied as biological triplicates are necessary for statistical support. Instead, read count data were used for the annotation of the tick transcriptome assemblies and GO enrichment analysis through identification of correct transcripts and their comparison among individual assemblies. The read count estimation was performed using RSEM (RNA-Seq by expectation-maximization) [49] implemented in the Trinity Transcript Quantification pipeline (accessed on 15 October 2019, [39]⁠). The contigs with low mapping counts were removed in order to avoid the presence of chimeric, fragmented, or biologically insignificant transcripts (cpm = 2 cut-off). BUSCO v3.0.2 search [42]⁠ was performed with the cpm = 2 filtered dataset and compared with BUSCO search performed with unfiltered transcriptome described in the “Transcriptome assembly and annotation” section. The mapping counts of each transcriptome were cross-sample normalized to the reference gene counts in order to identify transcripts that are present and specific for each transcriptome. The reference gene scaling factor was calculated using 5 normalization methods: TMM (trimmed mean of M-values), TMMwsp (trimmed mean of M-values with singleton pairing), UQ (upper quartile), and RLE (relative log expression) implemented in *edgeR* v3.12.0 as suggested by [50]⁠ and median normalization (MED) implemented in *edgeR* as described in [51]⁠. The reference genes’ read mapping counts were retrieved from the scaled matrices and their geometric means and geometric standard deviations were calculated in order to select the most efficient normalization method.

### Contig clustering and enrichment analysis

The counts of mapped reads in each transcriptome were transformed into a log_2_-scaled matrix in order to perform an enrichment analysis. Library-specific transcripts were inferred by hierarchical clustering of the Morpheus matrix analysis software (https://software.broadinstitute.org/morpheus/, last accessed on 30 November 2019) using complete linkage and 1-Pearson correlation metrics. Based on the resulting dendrogram, transcripts were assigned to clusters that are represented in one or more transcriptome assemblies; 2–50 clusters were built and a custom Python3 script was used to calculate within-cluster read count variability as a sum of squares function of Euclidean distances from respective cluster centroids. The frozen code is available on GitHub (Fussy, Z. (2019), GitHub repository, https://github.com/morpholino/PYTHON/blob/master/clustering_metrics_kliste.py). We used the elbow method to find the inflexion point where a minimal number of clusters explains ~90% of the total variability of the complete read count matrix. The transcripts assigned to each library were then subjected to GO enrichment analysis by goa-tools v0.8.12 [52]⁠ with Benjamini/Hochberg false discovery rate correction; the GO terms relationship file (go-basic.obo) from geneontology.org was last accessed on 30 November 2019. A custom Python3 script was used to visualize the enrichment inferred for each transcriptome assembly.

## Results

### Transcriptome assembly and annotation

A total of 430,604,850 paired-end 50-bp reads were obtained by sequencing of all five *I. ricinus* life stages. Trimming and quality filtering yielded 428,514,142 reads (see Additional file 1: Table S1 for details). The transcriptome assembly with Trinity assembler produced 117,583 “Trinity isoforms” and 83,534 “Trinity genes”. According to samtools flagstat, the Bowtie2 mapping rate of all libraries was around 80%, which corresponds to good quality assemblies with little information being lost in unmapped reads (see quality and mapping statistics available in Additional file 2: Table S2).

The RSEM package was used to distribute mapped reads among the five transcriptome assemblies of *I. ricinus.* Reads with low mapping counts (cpm = 2 threshold) were removed from the dataset of mapped reads as potentially misassembled or chimaeric. The number of transcripts after this low count filtering dropped from 83,534 to 25,872, which roughly corresponds to the genome assembly report of the related tick species, *I. scapularis* (23,340 transcripts) [53]⁠.

Read mapping counts were calculated in order to identify transcripts that are library-specific, demonstrating the presence or absence of individual transcripts across life stages. This does not necessitate biological replicates compared to statistical analyses required by quantitative RNAseq pipelines.

To enforce a more robust comparison, we scaled the five life-stage libraries to reference transcript counts selected through quantitative reference gene validation (see “Reference gene validation” section below).

Of the five different normalization techniques employed, the RLE method showed the lowest dispersion of reference gene variability among the five assemblies (see Additional file 2: Table S2.). The RLE was used to calculate reference gene normalization factor using mapping counts of five most stable reference genes of intermediate read mapping counts (*rps4*, *RpL32*, *rpl4*, *ferritin* and *RpL13A*), to which the matrix of read mapping counts of the five life stages was scaled.

Of 25,872 contigs of the pooled transcriptome assembly, whose quality and biological relevance was supported by a high number of mapped reads (i.e. contigs passing the cpm = 2 threshold), we could find annotation for 13,626 using InterProScan and 9510 of these were assigned GO terms. For some of these, and additional 653 contigs, we could find annotation using BLAST against the *I. ricinus* proteome deposited at UniProt, totalling 14,279 and 11,282 contigs with IPS and/or GO terms, respectively.

A BUSCO search of Arthropoda conserved orthologues within the unfiltered 83,534-contig assembly as an input reported 95.7% completeness. The assembly of contigs passing the cpm = 2 threshold exhibited 95.6 % completeness, which shows that cpm = 2 filtering effectively removed low-quality, fragmented, or chimeric contigs introducing false information or carrying an insignificant biological role for the *I. ricinus* assembly. A detailed BUSCO report is given in Additional file 3: Table S3.

Whole-body transcriptomes may lack mRNAs of lowly expressed genes. This concern motivated us to perform an additional verification of the comprehensiveness of our assembly. Our test was based on an assumption that genes that exhibit tissue-specific expression can be expressed in minute abundances compared to the genes expressed constitutively and organism-wide. Thus, their expression can be undetected if sequencing depth is insufficient or the assembly is of poor quality. We searched publications dealing with *I. ricinus* tissue-specific expression and selected randomly 3 tissue-specific genes and their paralogues. The list of sequences, their accession numbers, and corresponding publications showing their tissue-specific expression profile in *I. ricinus* are provided in Table 1. In particular, we chose 3 paralogues of cathepsin D (IrCDs) with expression restricted to tick midgut [54], a single sequence of the family of tick serine protease inhibitors (serpins) identified in *I. ricinus* salivary glands (Iris) [55], and two paralogues of fibrinogen-related protein, ixoderin A and ixoderin B showing tissue-specific expression profiles. Expression of ixoderin A was detected in haemocytes, salivary glands and midgut, and transcripts of ixoderin B were found in salivary gland tissue only [56]. Thus, a higher abundance and consequently a higher chance of full recovery of ixoderin A transcript was expected in comparison to ixoderin B. Importantly, our assembly contains sequences corresponding to all six queries (see Additional files 4, 5, 6, 7, 8 and 9 for alignments). We found four isoforms of Iris; all four sequences showed similarly high sequence identity. Of note, for the salivary gland-specific paralogue ixoderin B we also found a transcript, albeit 5’ truncated. Intriguingly, the ixoderin B query and hit showed only 70% identity on the amino acid level. However, the best hits within the *I. ricinus*-specific nr and tsa_nr databases (last accessed 11 Jun 2020) also returned best hits of 75% (GenBank: ABO09954.1) and 89% (GenBank: JAB75084.1) identity, respectively, both annotated as ixoderin B5. Nucleotide sequences of these two hits were included in the ixoderin B alignment to support the identity of the putative ixoderin B5 sequence from our assembly.

This transcriptome shotgun assembly project has been deposited at DDBJ/EMBL/GenBank under the accession GIDG00000000. The version described in this article is the first version, GIDG01000000.

### Reference gene validation

The selection of candidate reference genes for validation should be performed carefully, ideally based on existing data. Within Acari, only 4 species were analysed for reference gene selection and their validation: *Tetranychus urticae* [57]⁠, *Ripicephalus microplus*, *R. appendiculatus* [58]⁠, and *I. scapularis* [59]⁠. Our study focused on the identification of transcripts in tick life stages with recommendations from previous publications [58, 59] (Table 2). In total, 13 reference genes were selected for testing; *EF1ɑ*, *ferritin*, *GAPDH*, *H3F3A*, *ppiA*, *RpL32*, *RpL13a*, *rpl4*, *rps4*, *sdha*, *TBP*, *TUBB* and *v-ATPase*. Of these, *sdha* (succinate dehydrogenase complex flavoprotein subunit A) and *TBP* (TATA-box binding protein) were excluded from further analysis due to non-specific amplification during qRT-PCR assay optimization (data not shown). The best BLAST hits in our *I. ricinus* transcriptome assembly, using published *I. scapularis* references as queries, were used for primer design. These sequences were manually annotated and uploaded to GenBank. The list of *I. ricinus* reference genes used in reference gene validation assay, their accession numbers, and their respective *I. scapularis* queries with references are given in Table 2.

**Table 2:** Genes used in reference gene validation assay. The genes were selected based on validation in the referenced studies

Gene	Annotation	Reference	Query sequence	*I. ricinus* homologue sequence
*EF1ɑ*	Elongation factor 1α	[57, 58]	GU074814.1	MN728895
*ferritin*	Ferritin (somatic)	[112]	AY277906.1	MN728896
*GAPDH*	Glyceraldehyde 3-phosphate dehydrogenase	[57–59]	XM_002434302.1	MN728904
*H3F3A*	H3 histone family 3A	[58]	XM_002399526.1	MN728905
*RpL13A*	Ribosomal protein L13A	[59]	XM_002436237.1	MN728901
*ppiA*	Cyclophilin-type peptidylprolyl cis-trans isomerase A	[58]	XM_002407873.1	MN728903
*RpL32*	Ribosomal protein L32	[57]	XM_002399465.1	MN728898
*rpl4*	Ribosomal protein L4	[58]	XM_002402278.1	MN728900
*rps4*	Ribosomal protein S4	[59]	DQ066214.1	MN728897
*sdha*	Succinate dehydrogenase complex subunit A	[57]	XM_002408156.1	MN879329
*TBP*	TATA-box binding protein	[58, 59]	XM_002402081.1	MN879330
*TUBB*	β-tubulin	[57, 58]	GQ411364	MN728899
*v-ATPase*	Vacuolar-type H[+]-ATPase	[57]	XM_002413524.1	MN728902

**Fig. 1:**
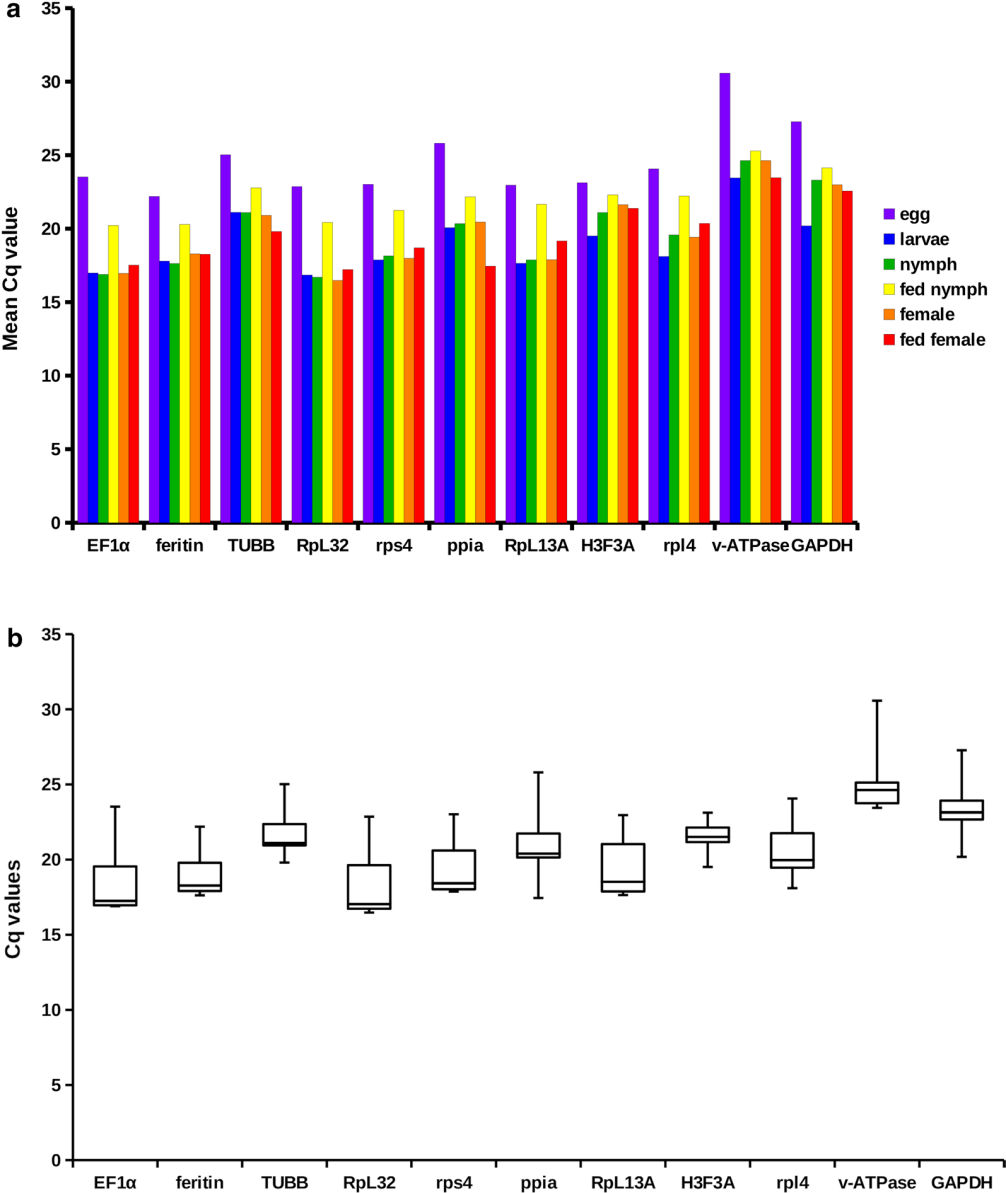
Reference gene expression levels and stability in different life stages. **a** Comparison of abundances of reference gene transcripts in six *I. ricinus* life stages. Each bar represents the geometric mean of Cq values calculated from biological triplicate. **b** Transcript abundance variation of each reference gene calculated for six *I. ricinus* life stages. Individual box plot elements represent the following variables: line, median; boxes, upper and lower quartile; whiskers, range of the dataset

The quantification of *I. ricinus* candidate reference gene expression was performed using qRT-PCR assay. The expression levels of reference genes and their variation in each life stage are given in Fig. 1a and b, respectively. The Cq values of each reference gene for each life stage and their biological replicates were integrated in BestKeeper v.1 [47]. The list of reference genes and their pairwise correlation coefficients (*r*) are listed in Table 3. All of the genes showed a strong positive correlation (*r*) ranging between 0.85–0.99. Moreover, the first eight genes in Table 3 showed a very strong positive correlation with an *r*-value above 0.9.

**Table 3:** Genes used in reference gene validation assay. Genes are ordered according to their pairwise correlation coefficient (*r*) in descending order

Rank	Gene	Pairwise correlation coefficient (*r*)	*P*-value
1	*rps4*	0.98500	0.00100
2	*RpL32*	0.98333	0.00100
3	*EF1ɑ*	0.98333	0.00105
4	*ferritin*	0.96467	0.00233
5	*rpl4*	0.95067	0.00447
6	*RpL13A*	0.94867	0.00523
7	*v-ATPase*	0.94133	0.00667
8	*TUBB*	0.93400	0.00939
9	*ppiA*	0.89333	0.01930
10	*GAPDH*	0.86467	0.03381
11	*H3F3A*	0.85867	0.04036

The pairwise correlation coefficient of all eleven tested reference genes indicates a high positive correlation. For normalization, we selected only the most stable reference genes as recommended previously [48, 60–62]⁠.

Furthermore, we excluded *EF1ɑ* gene from the calculation of HK factor for being a highly abundant transcript (8–12K cpm) (data not shown). Medium to highly abundant transcripts are more suitable reference genes due to their clear and reliable detection in every sample [63], whereas highly abundant transcripts tend to express less stably and thus their selection as reference genes should be made with precaution [64]⁠.

### Identification of transcripts specific for *Ixodes ricinus* life stages and Gene Ontology enrichment analysis

**Fig. 2:**
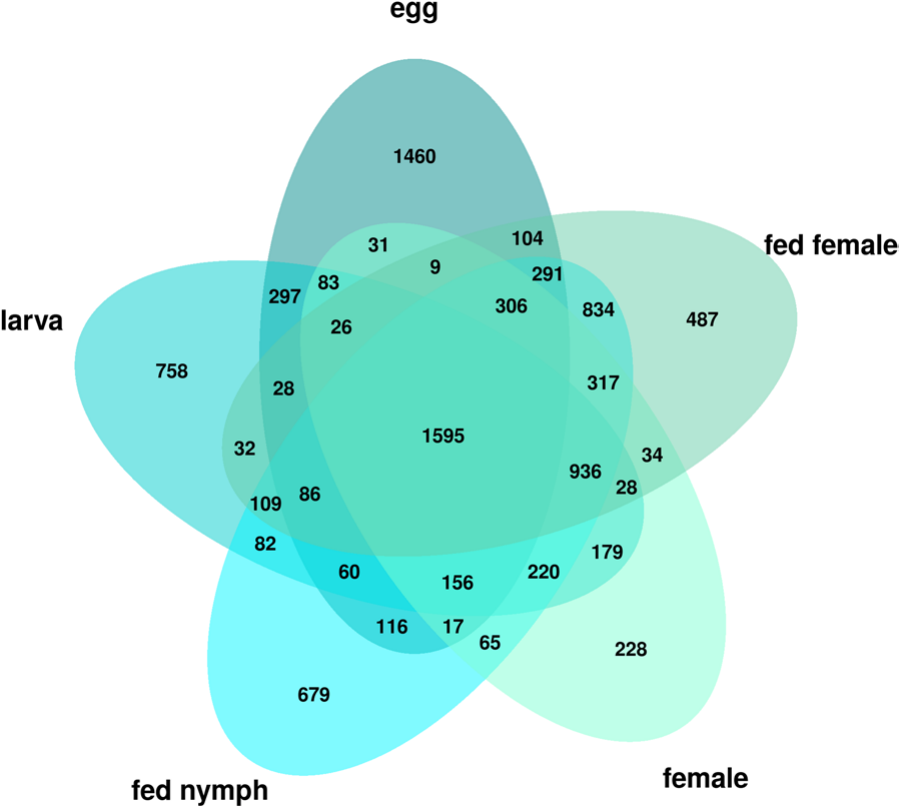
Graphical illustration of transcripts that are present in different transcriptome libraries. The diagram also shows the number of “housekeeping transcripts” present in all transcriptomes in the middle intersections. The numbers in the outer intersections show transcripts that are common for more but not all libraries

**Fig. 3:**
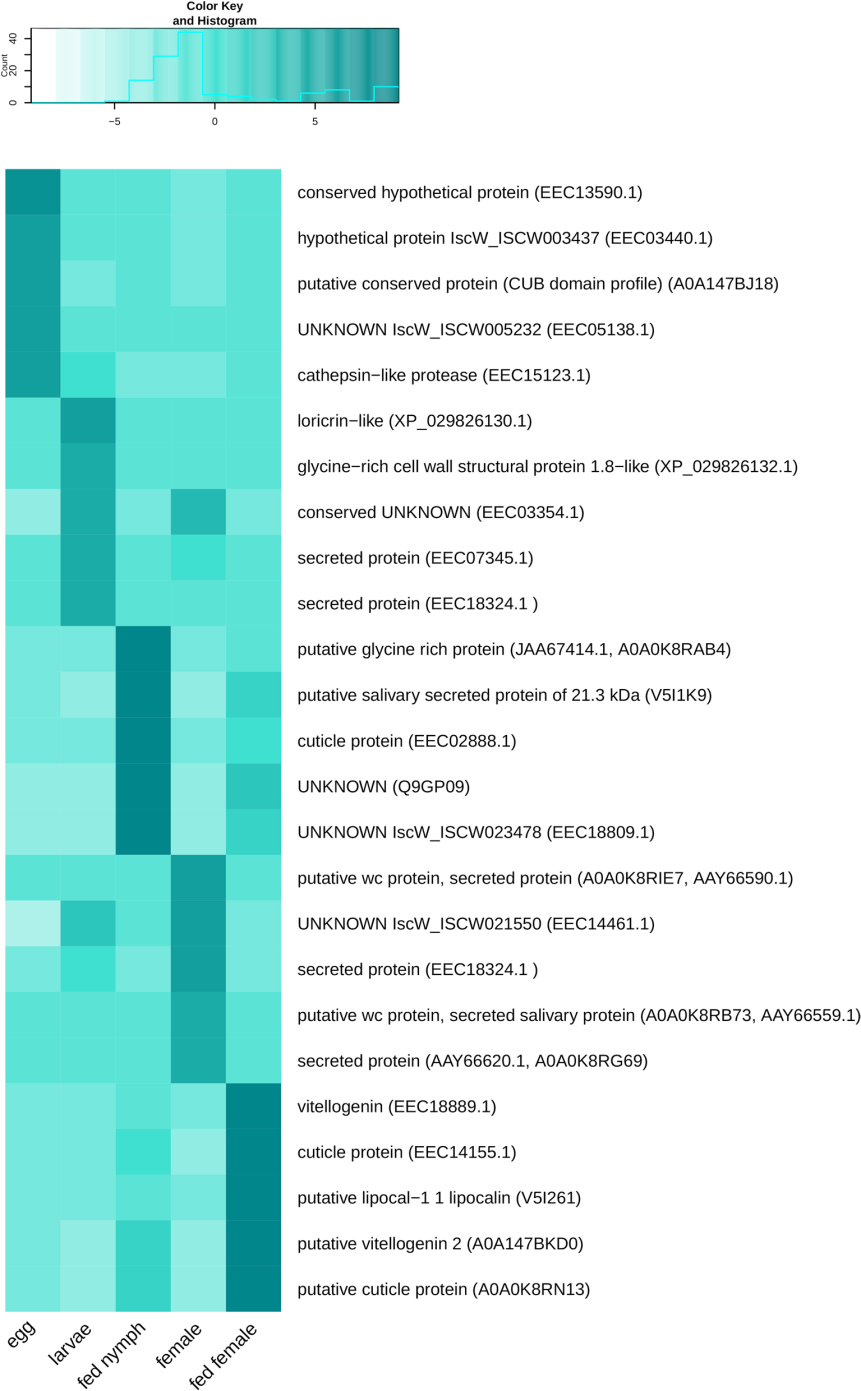
The presence of Top 5 transcripts in the *I. ricinus* common assembly used for GO enrichment and annotation. The heatmap is clustered according to transcripts read mapping counts in cpm in each life stage in descending order. Transcript descriptions are supplied with an accession number of the best BLAST hit in brackets

A Pearson correlation matrix assigning the transcripts to one or more of the transcriptome datasets was constructed. Of 25,872 transcripts, 10,266 were identified as life stage-specific. The remaining transcripts, identified in all assemblies, were classified as “housekeeping genes”, important throughout tick life stages. These transcripts were removed from further analysis as developmentally non-specific (see “HK_transcripts” column in Additional file 10: Table S4). Additionally, transcripts supported with very low read mapping counts (counts per million (cpm) < 2) as by Pearson correlation analysis, were removed from individual libraries prior to the identification of stage-specific transcripts. Numbers of library-specific, as well as housekeeping transcripts upon low read mapping count filtering, are presented in Fig. 2. Note the high number of transcripts present in transcriptomes of both feeding stages and of the egg transcriptome assembly. Transcripts in each stage-specific library were organized according to the number of mapped reads in descending order (Additional file 10: Table S4) and five of the transcripts that were assigned the most reads in each library (Top 5 transcripts) were selected for a detailed annotation and characterisation (Table 3). The presence of these transcripts in each life stage is illustrated in Fig. 3. It is evident that transcripts present in fed nymphs are specific for feeding stages and thus can be also found in fed females. Similarly, transcripts identified in unfed females are also detectable in the transcriptome of unfed larval stages. The Top 5 transcripts in *I. ricinus* eggs, on the other hand, seem to be only present in the egg transcriptome as evident from the heatmap colour intensity in the remaining four libraries.

**Fig. 4:**
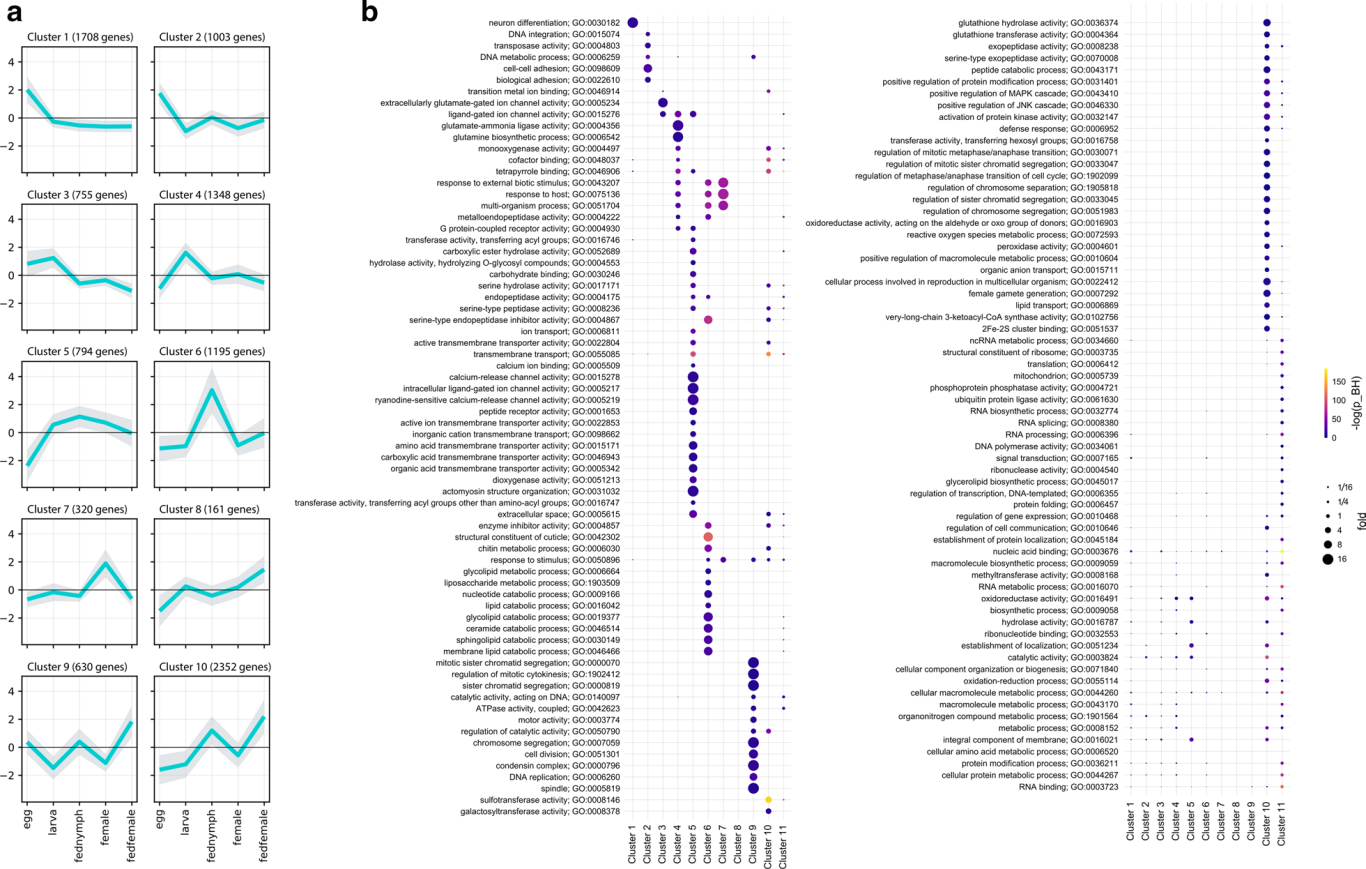
Illustration of stage-specific transcripts present in ten clusters identified with hierarchical clustering and intra-cluster variability evaluation. **a** The distribution of stage-specific transcripts within 10 transcript clusters. Mean log2-scaled read mapping counts are plotted per cluster and life stage, shades represent standard deviation. **b** Gene ontology terms enrichment for ten stage-specific clusters, including the cluster of “housekeeping gene” transcripts in the common transcriptome assembly of the five tick stage-specific libraries (Cluster 11). The colour and size of data points indicate − log10 values of Benjamini/Hochberg FDR-corrected *p*-values and fold enrichment compared to the whole set of transcripts, respectively

For the purposes of GO enrichment analysis and annotation, the matrix of read mapping counts of only the stage-specific transcripts was constructed and subjected to hierarchical clustering using complete linkage and 1-Pearson correlation metrics. The resulting dendrogram was cut to 2–50 clusters and their internal variability was calculated (see Methods). As per the elbow method, 10 clusters were the optimal number that accounted most efficiently for the total variability within the matrix (90.33% explained). Figure 4a presents the graphical illustration of the selected clusters of transcripts and shows their affiliation to particular stage-specific transcriptomes. Gene Ontology enrichment was determined for all transcript clusters, including an additional cluster of transcripts identified as “housekeeping genes”, which were removed prior clustering, and visualized as a categorized bubble plot (Fig. 4b). Of 10,266 stage-specific transcripts, 3839 were assigned a GO term; altogether, there were 964 unique GO terms, of which 112 are categorized to cellular components, 391 to biological processes, and 451 to molecular function. Transcripts present in all transcriptome assemblies with assignable GO terms (*n* = 7,443) were added for comparison (see cluster 11 of Fig. 4b). Figure 4b presents a detailed visualization of the enrichment of each GO term per stage and cluster.

### GO enrichment of transcript clusters in egg and larva life stages

Clusters 1–4 contain transcripts that were enriched only in egg and larva libraries. In particular, clusters 1 and 2 involve transcripts specific for egg development. Cluster 1 contained transcripts that are almost exclusively enriched in term GO:0030182 which entails the biological process “neuronal differentiation”. Cluster 2 was the most enriched with terms describing processes associated with DNA regulation and processing, possibly involving transposon activity, and with cellular adhesion. Cluster 3 presented transcripts identified in both egg and larva assemblies, enriched with GO terms describing transmembrane signal transduction *via* ligand-gated ion channels (GO:0005234 and GO:0015276). Transcripts in cluster 4 were identified in larval stage only and the most enriched GO terms in this cluster describe cellular energetic metabolism involving glutamine as a main source of energy (GO:0004356 and GO:0006542) or perhaps glutamatergic neuronal activity, in line with glutamate ion channel enrichment in cluster 3.

The annotation of the Top 5 transcripts in the egg identified the transcript c76180_g1, which is highly homologous to cathepsin-like protease (GenBank: EEC15123.1) that plays a major role in egg yolk degradation and is thus implicated in egg development [65]⁠. Another transcript found in the egg library (c80189_g5) is homologous to a conserved protein (GenBank: A0A147BJ18) containing an extracellular CUB domain (abbreviation derived from the complement C1r/C1s, Uegf, Bmp1), which is common for extracellular or cell membrane-associated proteins that are involved in developmentally regulated processes [66]⁠.

The Top 5 transcripts identified in larval transcriptome are related to salivary gland cement proteins as demonstrated by transcript c73350_g2 highly similar to loricrin-like protein (GenBank: XP_029826130.1) identified in *I. scapularis* [67, 68] and transcript sharing homology with cell wall components (c72525_g1 identical to GenBank: XP_029826132.1). The production of salivary gland related proteins in unfed stages implies an increased production of saliva, which is a typical physiological feature of questing unfed stages preparing for an upcoming blood meal especially after a prolonged period of starvation [69, 70]⁠.

### Fed nymph library-specific enrichment

Transcripts in cluster 5 and 6 were identified in fed nymph transcriptome; Cluster 5 aggregates transcripts that are present in fed nymphs, larvae and females. The processes identified for transcripts in this cluster extend a wide range of functions, playing a role in very diverse cellular functions and may underlie processes that have later developmental onset but are neglected in a feeding female where most effort and resources are put into blood meal processing and primarily into reproduction. The most dominant GO terms in this cluster describe cellular signalization *via* ligand-dependent calcium channels, transmembrane transporter activities (e.g. GO:0015278, GO:005217, GO:005219) or metabolic processes (e.g. GO:0030246, GO:0004175, GO:0017171) involving lipidic molecules and by extent cell membrane structure and integrity (e.g. GO:0052689, GO:0016746), consistent with the observation in larvae (see below). Cluster 6 presents transcripts enriched in fed nymphs only. The most conspicuous activities associated with this cluster are involved in cuticle formation, catabolism of lipids, and enzymatic inhibition (e.g. GO:0042302, GO:0019377, GO:0004867). These activities are directly linked to tick feeding resulting in the fast growth of a tick cuticle-covered body which secures an efficient blood meal accommodation and also to blood digestion and its regulation, respectively. Additionally, the signalling pathways, responding to a foreign organism, are also activated in feeding nymphs (e.g. GO:0075136, GO:0043207).

### GO enrichment of unfed female

Cluster 7 comprised transcripts enriched in unfed females. The GO terms typical for this cluster describe cofactor/tetrapyrrole binding activity (GO:0048037, GO:0046906) and signalling pathways triggered by external biotic stimuli or host (GO:0043207, GO: 0075136, GO:0051704).

The enrichment of these terms also occurred in larvae and fed nymphs, however, to a lower extent than in unfed females. Two of the Top 5 transcripts identified in the female library (c69267_g3 and c69267_g2) are homologous to salivary gland wc proteins identified previously in both *I. ricinus* and *I. scapularis* (GenBank: A0A0K8RIE7/AAZ66590.1 and A0A0K8RB73/AAY66559.1, respectively).

### Fed female library-specific enrichment

Transcript clusters 8 and 9 were specific for the fed female life stage. Interestingly, no enrichment occurred in cluster 8, possibly as it is the smallest transcript cluster (161 contigs, i.e. 1.57% of transcripts assigned to different stage-specific libraries) and none of the class of transcripts was large enough to enable enrichment. Cluster 9 accommodates transcripts whose functions, as by enrichment, are nearly exclusively related to cell division processes including transcripts driving mitosis, DNA replication, chromatid segregation, etc. (e.g. GO:0007059, GO:0006260, GO:0005819).

### Feeding responsive transcript clusters

Cluster 10 included transcripts that are typical for both, fed nymphs and fed females, and extends the widest range of GO terms of all ten transcript clusters. The most enriched terms describe processes which feeding stages utilize for blood meal processing and regulation, including protease activity, lipid processing and transport, also comprising lipid metabolism-related to cell wall integrity and composition (e.g. GO:0070008, GO:0036374, GO:0102756, GO:0006869). Blood-feeding is also associated with activities that are required for processing of toxic products of blood meal degradation (GO:0004601, GO:0072593) and for the elimination of foreign molecules and potentially pathogens incoming with the blood meal, thus inducing immune defence pathways (GO:0006952). Similarly to cluster 9, the transcripts associated with mitosis, cell division, and related processes are enriched (e.g. GO:0030071, GO:0033047, GO:0033045). Also related are terms enriched for transcripts underlying factors regulating cell cycle (GO:0043410, GO:0046330). Another group of related GO terms describe transcripts influencing ovarian development, gametogenesis and reproduction (GO:0022412, GO:0007292). The Top 5 transcripts identified in both libraries of fed stages are mainly related to salivary gland-related functions triggered during feeding and cuticle protein synthesis to support blood meal accommodation within the tick. For example, transcript c81210_g1 shows high similarity to the sequence (GenBank: Q9GP09) described in a study of salivary gland factors and their expression induced by blood meal in *I. ricinus* [71]⁠. Further characterisation of this transcript showed its high sequence identity (over 80%) with a glycine-rich protein identified in *I. scapularis*, related to fibroin heavy chain protein (GenBank: XP_029833024) involved in a build-up of cement which the tick uses for attachment of its hypostome to the host during feeding [68]. Similarly, the remaining annotated transcripts present in both libraries of feeding stages are of salivary gland origin or cuticle-related. Additionally, fed females of haematophagous parasites typically highly expressed transcripts related to reproduction. This corresponds to the presence of transcripts homologous to vitellogenins (c79317_g2 identical to GenBank: EEC18889.1, and c80433_g1 identical to GenBank: A0A147BKD0), expressed in egg yolk, that were found among the Top 5 transcripts in the fed female library.

Also present in fed female transcriptome is a transcript homologous to lipocalin 1, which is a protein typically produced in tick salivary glands and is a tick-specific evolutionary adaptation to blood-feeding [72]⁠.

The transcripts typical for both unfed larvae and females are mainly related to the processes involved in cell growth and cell cycle control or metabolism. An example is transcript c72929_g1 highly similar to the *I. scapularis* secreted protein (GenBank: EEC07345) of unknown identity whose homologous transcript GenBank: XP_029841102.1 underlies the production of a conserved cwc2 group pre-mRNA-splicing factor. Another two transcripts (c82554_g1 and c79150_g3, homologous to *I. scapularis* EEC03354 and EEC14461, respectively) of unknown function each contain an acyltransferase 3 family domain and an NRF superfamily domain (related GO term GO:0016747). The activity of proteins containing both these domains is usually associated with lipid metabolism, processing, or transport and could be, by extent, also related to cell membrane structure and integrity and cell membrane functional components, which was also observed in the model organism *Caenorhabditis elegans* [73, 74]⁠.

## Discussion

### Feeding-responsive transcripts

Four transcript clusters (6, 8, 9 and 10) recovered from our analysis are specific for fed stages of *I. ricinus*. Functional classification of most typical transcription was linked to cuticle formation and chitin metabolism as well as to the activation of blood-meal processing enzymes, all in response to a blood meal. These findings were anticipated and are in line with previous observations [30, 31, 33, 75].

Factors involved in mitosis and cell division are also commonly seen in feeding stages. Specific expression of these factors reflects the precocious cellular growth occurring shortly after a blood meal. Consistently, two GO terms (GO:0022412 and GO:0007292) involved in reproduction are enriched in both feeding stages. Their association with feeding females is fairly straightforward, underlying factors driving ovarian development and gamete generation. Enrichment of these transcripts in feeding nymphs is not self-evident; however, a lead to its functional elucidation in this non-adult stage can be found in thinly documented ecdysteroid hormonal regulation in ticks. The regulation of ecdysis is essential for the proper development of all arthropods, underlined by the existence of an organ dedicated to ecdysteroids production. In chelicerates, ecdysteroidogenesis and its regulation is poorly understood but possibly restricted to ovarian tissue in both its mature and immature life stages as in the soft tick *Ornithodoros moubata* [76]⁠. This study suggests that factors driving reproduction, ovary development, and ecdysis might be tightly regulated. The involvement of tick nymphal ovary in processes decoupled from reproduction is demonstrated by its insensitivity to vitellogenin which plays a principal role in egg yolk formation and thus in gametogenesis and reproduction in arthropods [77, 78]⁠. Collectively, these findings along with an enrichment of reproductive transcripts in our fed nymph specific library strongly support the importance of ovary for ecdysteroidogenesis in ticks, thus corroborating the enrichment of reproduction-related transcripts in the feeding nymph. We suggest that transcripts functionally linked to reproduction in the library of fed nymphs are in fact involved in the development of ovary which clearly plays a role in the regulation of moulting in other ticks, and to the best of our knowledge, our findings are the first to describe this connection in *I. ricinus* tick.

### Transcripts identified in unfed stages

The transcripts enriched in basic metabolic processes are present in larvae (glutamine-based metabolism; GO:0005234, GO:0004356, GO:0006542). Most of the related processes are also active in other life stages but their transcription is presumably overshadowed by processes more relevant for these stages. However, we suggest a linkage of some of these seemingly basic processes to specific functions in unfed tick stages. Perhaps, glutamate-ammonia ligase activity may point out GABA-ergic neuronal activity promoting larval motility [79]⁠. Response to host or to external biotic stimuli was present in both females and larvae, though host-induced pathways were also observed for fed nymphs and thus might not be exclusive to unfed stages [80–83]⁠. The presence of host response transcripts in larvae seems to be consistent with transcripts underlying glutamine metabolism of GABA-ergic neurons and host-seeking and was also observed in an unfed larva transcriptome library in other ticks [84]⁠.

Transcripts highly similar to *I. scapularis* secreted wc proteins were found prominent in unfed females (Table 4). This group of peptides, characterised by the presence of Trp-Cys dipeptide motif (thus “WC” proteins) at their C termini, are apparently specific to ticks and their function is yet to be elucidated [75, 85, 86]⁠.

**Table 4:** Highest-ranking transcripts of the read mapping counts matrix in each of the five stage-specific assemblies

Contig ID	Best blast hit	Description
Egg		
c77007_g1	EEC13590.1	Conserved hypothetical protein
c80053_g1	EEC03440.1	Hypothetical protein IscW_ISCW003437
c80189_g5	A0A147BJ18 (IPR000859)	Putative conserved protein (CUB domain profile)
c77756_g1	EEC05138.1	Unknown IscW_ISCW005232
c76180_g1	EEC15123.1(GO:0006508; GO:0008234; IPR000169)	Cathepsin-like protease (proteolysis; cysteine-type peptidase activity; eukaryotic thiol (cysteine) proteases asparagine active site)
Larva		
c73350_g2	XP_029826130.1 (PR01217)	Loricrin-like (LOC115311581) (proline-rich extensin signature)
c72525_g1	XP_029826132.1 (PR01228)	Glycine-rich cell wall structural protein 1.8-like (eggshell protein signature)
c82554_g1	EEC03354.1 (GO:0016747; IPR002656)	Conserved unknown (transferase activity, transferring acyl groups other than amino-acyl groups; acyltransferase family)
c72929_g1	EEC07345.1	Secreted protein
c69133_g1	EEC18324.1	Secreted protein
Fed nymph		
c23277_g1	JAA67414.1, A0A0K8RAB4 (PR01217)	Putative glycine rich protein (proline-rich extensin signature)
c80070_g2	V5I1K9	Putative salivary secreted protein of 21.3 kDa (fragment)
c78019_g4	EEC02888.1 (GO:0042302; IPR000618)	Cuticle protein (structural constituent of cuticle; chitin-binding type R&R domain profile; insect cuticle protein)
c81210_g1	Q9GP09	Unknown
c75846_g6	EEC18809.1 (PR01217)	Unknown IscW_ISCW023478 (proline-rich extensin signature)
Female		
c69267_g3	A0A0K8RIE7 (AAY66590.1)	Putative wc protein, secreted protein
c79150_g3	EEC14461.1	Unknown IscW_ISCW021550
c75091_g1	EEC18324.1	Secreted protein
c69267_g2	A0A0K8RB73 (AAY66559.1)	Putative wc protein, secreted salivary protein
c1565_g1	AAY66620.1, A0A0K8RG69	Secreted protein
Fed female		
c79317_g2	EEC18889.1 (GO:0005319; GO:0006869; IPR001747; IPR001846)	Vitellogenin (lipid transporter activity; lipid transport; VWFD domain profile; von Willebrand factor type D domain; vitellogenin domain profile)
c79647_g1	EEC14155.1 (GO:0042302; IPR000618)	Cuticle protein (structural constituent of cuticle; Chitin-binding type R&R domain profile; insect cuticle protein)
c73798_g1	V5I261 (GO:0030682; GO:0043176; IPR002970)	Putative lipocal-1 1 lipocalin (Fragment) (evasion or tolerance of host defenses; amine binding; tick histamine binding protein)
c80433_g1	A0A147BKD0 (GO:0005319; GO:0006869; IPR001747; IPR001846)	Putative vitellogenin 2 (Fragment) (lipid transporter activity; lipid transport; VWFD domain profile; von Willebrand factor type D domain; vitellogenin domain profile)
c74644_g1	A0A0K8RN13 (GO:0042302; IPR000618)	Putative cuticle protein (fragment) (structural constituent of cuticle; chitin-binding type R&R domain profile; insect cuticle protein)

*Notes*: Accession numbers of the best blast hits and their description are supplied. GO term and/or IPS annotations including their respective descriptions are also listed when available

### Transcription specific for egg development and tick embryogenesis

Despite numerous efforts in basic and applied tick research [87–91], only one study has concentrated on the early stages of tick development [92]⁠. A paucity of early-stage information hinders the implementation of targeted approaches, such as RNA interference or characterisation of vaccine candidates [93, 94]⁠. Our study is among the first to provide a comprehensive and biologically relevant catalogue of transcripts for future research aiming at controlling the population of ticks in the early stages of their development.

Apart from an anticipated functional enrichment in neuronal development (GO:0030182), observed in the embryonic development of other arthropods as well [95]⁠, cell adhesion-related transcripts (GO:0098609), clearly important for embryonic morphogenesis, were also highly enriched [96]⁠.

Of interest was an enrichment of factors unleashing mobile DNA transposition and consequent DNA integration which are typically reactivated during embryonic development. An elevated expression of transposase associated with transposon activity is usually less regulated in order to enable an implementation of developmental regulation of host DNA during embryogenesis [97]⁠. Transposition-related activities can thus be considered markers of early tick embryonic development.

An increased expression of cathepsin protease observed in the egg library was also anticipated as cathepsin proteases take part in egg yolk proteolysis and thus are crucial for the energetic metabolism during embryogenesis [65, 94, 98, 99]⁠. Consistently, cathepsins have been in the scope as targets for tick control [100, 101]⁠. CUB domain proteins were also found among egg-specific transcripts and their expression in an egg is most typically manifested in embryonic developmental factors [66]⁠. CUB domains are found conserved in Metazoa and most often occur in cell surface proteins that mediate interactions of embryonal morphogenetic proteins and metalloproteases [102–104]⁠.

### Reference gene validation assay

Reference gene validation using qRT-PCR is an essential step for proper quantitative data normalization. Currently, this is the most recognized methodology to provide reliable gene expression comparisons [105]⁠. It is also a method of choice in cross-sample normalization of read count data or for the evaluation of normalization techniques in studies dealing with transcriptomic NGS data [50, 106, 107].

All genes tested in our study showed a high degree of stability among tick life stages as indicated by the correlation coefficient (*r*). Still, it is crucial to carefully select candidate reference genes with respect to specific physiological circumstances given by the experimental design. Related to the tick development, specific examples can be found in haematophagous arthropods experiencing one or more blood meals in their life-cycle. This represents a radical alteration of physiological conditions and profoundly affects the expression of many genes, including those that are considered fairly stable in a majority of other organisms as is the case of β-actin in the kissing bug *Rhodnius prolixus* [108] or ribosomal components of *Ae. aegypti* during vitellogenesis triggered by a blood meal [109]⁠. In our study, several ribosomal genes (*RpL13A*, *RpL32*, *rpl4* and *rps4*) were tested and all of them proved highly stable. We ascribe such discordance to fundamental differences in the life-cycle among ticks and mosquitoes. In particular, the duration of blood-feeding is substantially different, taking approximately two minutes in the mosquito in comparison to several days in the tick [77, 110]⁠, which certainly has implications to the initiation and progress of vitellogenesis [22, 76, 111] and hence the expression stability of ribosomal genes. Accordingly, inspection of partially fed stages may have also contributed to ribosomal transcript stability in this work. Thus, the set of reference genes, rather than just one of them, can be applied in future studies comparing gene expression between tick life stages. However, the robustness of our reference gene set can lower under different study designs, for instance monitoring gene expression under specific experimental conditions, tissue-specific expression, or time-lapse expression in time points of tick development different than here, such as fully fed stages. We, therefore, highlight the importance of *de novo* validation of selected reference genes for specific experimental designs.

### Cataloguing a stage-specific transcription of *Ixodes ricinus*

The main mission of our project was to produce a comprehensive catalogue of transcripts of the tick *I. ricinus* and identify transcripts specific for developmental stages of interest by their cross-stage comparison. Using entire bodies for sequencing was thus of importance. This approach allowed us to collect all transcripts that are typical of each life stage. The main limitation here is the risk of losing information about transcripts of very low abundance whose expression is either tissue-specific, or is restricted to a short period during development, or is induced by a specific physiological state. Sequencing of transcriptomes derived from individual tissues or time points, on the other hand, suffers from compositional bias which is manifested in the overrepresentation of functionally specific populations of transcripts, disregarding expression in other tissues or developmental periods. This is evident from the identification of transcripts exclusive for RNA libraries originating in different life stages of *I. ricinus* salivary glands [31]⁠.

In order to detect low-abundance transcripts, we performed sequencing of each library to a very high read depth using the HiSeq2000 Illumina platform. For each of our libraries, we received 72–120 M of reads (PE50). Additionally, we prepared our transcriptome assembly from reads combined from all five stage-specific libraries. This approach should secure an even more comprehensive catalogue of transcripts representing the overall coding potential of the *I. ricinus* genome. Comparison of transcript number in our assembly (25,872) with the coding capacity of *I. scapularis* (23,340) [53], the closest relative species with publicly available genome data, also provided an indication about the high completeness of our assembly.

We additionally confirmed a good recovery of low-abundance transcripts in our assembly by searching for transcripts of genes whose expression was found to be tissue-specific and thus presumably very low in the population of all transcripts of the whole-body transcriptome. Based on previously published studies, we selected five genes whose expression had been found restricted to a specific tissue in the tick. Our BLAST search yielded 100% of query transcripts, which again validates the high completeness of our *I. ricinus* assembly. Evaluation of another tissue-specific marker, however, revealed that the sequence of the putative ixoderin B is 5’ partial. Ixoderin B is a salivary gland-specific transcript in contrast to its A isoform which exhibits organism-wide transcription and whose corresponding transcript was fully assembled in our transcriptome. We admit limitations to our dataset in terms of both transcript presence and CDS completeness and encourage to inquire public databases to recover complete sequences of lowly expressed genes in the future. It is however very likely that the majority of low-abundance transcripts we removed from the raw assembly of 83,534 sequences are truncated sequences which do not represent biologically relevant transcription of the interrogated life stage. Our dataset thus constitutes a representative transcriptome of *I. ricinus* across life stages and the removal of truncated low-quality transcripts does not negatively influence an estimation of stage-specific transcription.

Our study was designed to cover the interpopulation expression variability of *I. ricinus* without losing the chance of recovery of lowly expressed transcripts or rare transcript isoforms underlying an interpopulation expression variability of this species. We thus pooled triplicated samples collected from ticks originating in different tick populations. This allowed us to produce a high per sample read coverage without compromising the full assembly of rare transcripts. This was done on the expense of reducing the reliability of transcriptome quality evaluation and general significance of conclusions that we derive from our sequencing project. However, with a series of quality filtering and evaluation tests as for example the BUSCO assessment, we were able to present a list of indicators supporting high efficiency of our bioinformatic approach and good quality of the output data. The same experimental design was also presented in previous publications where a single library per sample was sequenced on the expense of high sequence coverage [31, 33]. Using well-established bioinformatic pipelines along with a number of quality evaluation tests, the authors were able to construct reliable time- and tissue-dependent tick transcriptome assemblies supported by a single sequencing library per sample. This facilitated a postulation of firm conclusions based on well-organized analyses focused on the proper characterization of particular sample-specific assemblies, their reciprocal comparison, and an inference and functional annotation of sample-specific transcription.

With precaution, we performed *de novo* assembly instead of reference driven transcriptome reconstruction. Despite the existence of annotated reference genome of *I. scapularis*, a genome reference of related species may have obstructed assembly of *I. ricinus* sequences transcribed from highly interspecifically variable loci.

Our study was also organized to produce a representative catalogue of stage-specific transcripts that do not provide strong quantitative information about gene expression. Our transcriptome assembly was however submitted to a series of quality testing, normalization, and functional annotations. The bioinformatic pipeline that was eventually applied to our sequencing data was used upon thorough consultation of existing studies dealing with stage-specific transcription and with general bioinformatic guidelines [48, 50, 84, 107]⁠. Due to a unique design of our study, we organized our work by a combination of different approaches based on assumptions derived from the type and nature of our data and, at the same time, from biological questions postulated in our project. The resulting outputs provided valuable information about processes and functions typical for each life stage. Moreover, our transcriptome database represents a priceless pool of information for initiation of future research projects that can be built upon the mere knowledge about transcription in *I. ricinus* followed by the associated validation of transcripts sequences and their cross-referencing with public databases. Moreover, the collection of transcripts specifically expressed in eggs can be exploited in the development of *in vitro* methods in tick-derived cell cultures originating in tick embryonic cells [38]⁠.

The descriptive nature of our study poses certain limits for an interpretation of our data in comparison with other quantitatively designed studies. On the other hand, putting our data into proper context, even within quantitative studies, can still present valid points for discussion with perspective for further confirmation by experimental approaches where required.

## Conclusions

Our work presents and discusses new findings about developmentally specific transcripts identified in *I. ricinus* eggs, larvae, partially fed nymphs, females, and partially fed females. Proper normalization of our transcriptome assemblies was performed using a set of reference genes whose stability was verified by a quantitative gene validation essay of eleven candidate reference genes also presented in this study. Our data confirm the identity of transcripts involved in tick feeding presented in previous studies and support the role of ovary in ecdysteroidogenesis also in *I. ricinus* as was previously suggested in related tick species. Additionally, we describe processes and specific transcripts apparently important for embryogenesis, whereby most conspicuously energetic metabolism, developmental mobile DNA reactivation, and subsequent initiation of morphogenetic processes are crucial. Our study presents new insights into early and late tick development, consistent with previously published research and draws our findings into new biological contexts. As a whole, our work extends the collection of important information for further investigation in both basic and applied tick research, including the development of tick-targeted population control programs.

## Supplementary information

**Additional file 1: Table S1.** Description of primers used in the housekeeping gene validation assay.

**Additional file 2: Table S2.** Summary of library mapping statistics and cross-sample normalization.

**Additional file 3: Table S3.** Summary of *de novo* assembly quality statistics.

**Additional file 4: Alignment S1.** Alignment of cathepsin D1 (GenBank: EF428204.1) query sequence and a corresponding transcript recovered from *Ixodes ricinus* stage-specific transcriptome assembly (c79321_g3_i1).**: Alignment S1.** Alignment of cathepsin D1 (GenBank: EF428204.1) query sequence and a corresponding transcript recovered from *Ixodes ricinus* stage-specific transcriptome assembly (c79321_g3_i1).

**Additional file 5: Alignment S2.** Alignment of cathepsin D2 (GenBank: HQ615697.1) query sequence and a corresponding transcript recovered from *Ixodes ricinus* stage-specific transcriptome assembly (c81800_g1_i2).

**Additional file 6: Alignment S3.** Alignment of cathepsin D3 (GenBank: HQ615698.1) query sequence and a corresponding transcript recovered from *Ixodes ricinus* stage-specific transcriptome assembly (c81927_g1_i1).

**Additional file 7: Alignment S4.** Alignment of Iris (GenBank: AJ269658.2) query sequence and four corresponding transcripts representing Trinity assembler isoforms recovered from *Ixodes ricinus* stage-specific transcriptome assembly (c83951_g1_i2, c83951_g1_i5, c83951_g1_i3, c83951_g1_i1).

**Additional file 8: Alignment S5.** Alignment of ixoderin A (GenBank: AY341424.1) query sequence and a corresponding transcript recovered from *Ixodes ricinus* stage-specific transcriptome assembly (c80994_g1_i1).

**Additional file 9: Alignment S6.** Alignment of ixoderin B (GenBank: AY341424.1) query sequence and a truncated corresponding transcript recovered from *Ixodes ricinus* stage-specific transcriptome assembly (c82323_g13_i1).

**Additional file 10: Table S4.** List of stage-specific and housekeeping transcripts.

## Data Availability

All data generated or analysed during this study are included in this published article and its additional files. The Transcriptome Shotgun Assembly project has been deposited at DDBJ/EMBL/GenBank under the accession GIDG00000000. The version described in this paper is the first version, GIDG01000000.

## Abbreviations

cpm: counts per million
geoSTDEV: geometric standard deviation
GO: Gene Ontology
MED: median normalization
RLE: relative log expression
RSEM: RNA-seq by expectation-maximization
TBEV: tick-borne encephalitis virus
TMM: trimmed mean of M-values
TMMwsp: trimmed mean of M-values with singleton pairing
UQ: upper quartile
