# Supplementary material for: Catalogue of stage-specific transcripts in Ixodes ricinus and their potential functions during the tick life-cycle
Source: Parasit Vectors. 2020 Jun 16;13:311. doi: 10.1186/s13071-020-04173-4 (PMC7296661; doi:10.1186/s13071-020-04173-4)
Supplement: Supplementary file 1 — Additional file 1: Table S1. Description of primers used in the housekeeping gene validation assay. [file 13071_2020_4173_MOESM1_ESM.docx]

**Additional file 1: Table S1.** Description of primers used in the housekeeping gene validation assay.

| **Gene** | **primer sequence (5’ → 3’)** | **amplicon size (bp)** |
| --- | --- | --- |
| *EF1ɑ* | F: CGCCTGGGTGTTGGACAAGCTGAA | 167 |
|  | R: CGCAGTCGGCCTGGGAGGTA |  |
| *ferritin* | F: TGTGCGACTTCCTGGAGGGC | 88 |
|  | R: ACACGCTTCAGGTTGGTCACG |  |
| *GAPDH* | F: CGTCTTCCAGGAGCTGAAGCC | 232 |
|  | R: CGAGGGTGGTGAAGACGCC |  |
| *H3F3A* | F: GCTCAAGACTTCAAGACGGACCTC | 103 |
|  | R: GGTTGGTGTCCTCAAACAGACCC |  |
| *RpL13A* | F: TCGCTAAGACACTCCTCCACGG | 109 |
|  | R: CGCAGGAACGACAGGTACTTCAG |  |
| *ppiA* | F: GAGTGCCCATTCTGCCTCTGC | 94 |
|  | R: GCTCCATCAGCAGTCACGTCG |  |
| *RpL32* | F: CGTCGCCGCTTCAAGGG | 188 |
|  | R: CGCTTTTTGGATGACACGCCG |  |
| *rpl4* | F: TGACCCGCAGAGCATCTGTG | 84 |
|  | R: GCTTGACGCCTCGCTTCTCAG |  |
| *rps4* | F: ATGGCACGGGGACCTAAGAAG | 75 |
|  | R: TCCACCGAGCTTGTCCAGC |  |
| *TUBB* | F: TCCAACACGACAGCCATTGCC | 114 |
|  | R: GCCTTCCTCCATGCCTTCACC |  |
| *v-ATPase* | F: ACGCTCTAGCCGTGTCAAGAGAC | 146 |
|  | R: GCCATCAGCCAGCACAAGG |  |
