# Supplementary material for: Catalogue of stage-specific transcripts in Ixodes ricinus and their potential functions during the tick life-cycle
Source: Parasit Vectors. 2020 Jun 16;13:311. doi: 10.1186/s13071-020-04173-4 (PMC7296661; doi:10.1186/s13071-020-04173-4)
Supplement: Supplementary file 3 — Additional file 3: Table S3. Summary of de novo assembly quality statistics. [file 13071_2020_4173_MOESM3_ESM.docx]

**Additional file 3: Table S3.** Summary of *de novo* assembly quality statistics.

| **BUSCO raw assembly** | **#** | **%** |
| --- | --- | --- |
| Complete BUSCOs | 1,021 | 95.7 |
| Complete and single-copy BUSCOs | 706 | 66.2 |
| Complete and duplicated BUSCOs | 315 | 29.5 |
| Fragmented BUSCOs | 26 | 2.4 |
| Missing BUSCOs | 19 | 1.9 |
| Total BUSCO groups searched | 1,066 | 100 |
| **BUSCO cpm2 assembly** | **#** | **%** |
| Complete BUSCOs | 1,019 | 95.6 |
| Complete and single-copy BUSCOs | 705 | 66.1 |
| Complete and duplicated BUSCOs | 314 | 29.5 |
| Fragmented BUSCOs | 22 | 2.1 |
| Missing BUSCOs | 25 | 2.3 |
| Total BUSCO groups searched | 1,066 | 100 |
