# Supplementary material for: Catalogue of stage-specific transcripts in Ixodes ricinus and their potential functions during the tick life-cycle
Source: Parasit Vectors. 2020 Jun 16;13:311. doi: 10.1186/s13071-020-04173-4 (PMC7296661; doi:10.1186/s13071-020-04173-4)
Supplement: Supplementary file 4 — Additional file 4: Alignment S1. Alignment of cathepsin D1 (GenBank: EF428204.1) query sequence and a corresponding transcript recovered from Ixodes ricinus stage-specific transcriptome assembly (c79321_g3_i1).: Alignment S1. Alignment of cathepsin D1 (GenBank: EF428204.1) query sequence and a corresponding transcript recovered from Ixodes ricinus stage-specific transcriptome assembly (c79321_g3_i1). [file 13071_2020_4173_MOESM4_ESM.docx]

**Additional file 4: Alignment S1.** Alignment of cathepsin D1 (GenBank: EF428204.1) query sequence and a corresponding transcript recovered from *Ixodes ricinus* stage-specific transcriptome assembly (c79321_g3_i1). Dots indicate agreements, hashes an absence of sequence in the alignment. Underlined sequence in the Consensus represent an ORF.

Consensus GAACAATCCAAACAAGCTCCAACAGTTTCCTATCTACGGTCAGCTCCTGAACTATCGTCG 60

EF428204.1 ------------------------------------------------------------

c79321_g3_i1 ............................................................ 60

Consensus GCCTCAGCCATGTAAAGCACGAGGCCGCTATCAATCGCATTGTGTGCGCCCGATAGGTCT 120

EF428204.1 ------------------------------------------------------------

c79321_g3_i1 ............................................................ 120

Consensus TCTCTATAAAACGTAACAGGAGGTTCGACCGCGACTACARATTGTACGTGCAGCAAAACG 180

EF428204.1 ------------------------------------...A.................... 24

c79321_g3_i1 .......................................G.................... 180

Consensus ASGCACGACGGTARAATGCGCTGTTCGACCTTAACTTTGACCCTTGTGGTCCTATTGGCC 240

EF428204.1 .G...........A.............................................. 84

c79321_g3_i1 .C...........G.............................................. 240

Consensus GCAGAATGTGCGTTTGGCGCTTTCAGGATCCCGCTCACGAGATTCAAGTCTGTGCGGAAG 300

EF428204.1 ............................................................ 144

c79321_g3_i1 ............................................................ 300

Consensus CAACTGGCGGAGGAAGGGATCTATATTCACGAAGGACCCTATCCGGAGCCACTGGTCAAC 360

EF428204.1 ............................................................ 204

c79321_g3_i1 ............................................................ 360

Consensus TTACTCGATGTGGAATACTACGGTCCCATAAGCATCGGGACCCCGCCCCAAGACTTCCAG 420

EF428204.1 ............................................................ 264

c79321_g3_i1 ............................................................ 420

Consensus GTGATTTTCGACACCGGTTCTGCTAACCTTTGGCTGCCGTCTTCGAAGTGCACGACAAAG 480

EF428204.1 ............................................................ 324

c79321_g3_i1 ............................................................ 480

Consensus TACTGTTTGCACCATCACAGATACGACAGCAGCAAATCCAGTACCTACGAAGCGGATGGT 540

EF428204.1 ............................................................ 384

c79321_g3_i1 ............................................................ 540

Consensus CGCAATTTCACCATAGTGTACGGATCAGGAAATGTCGAAGGATTTATCAGCAAAGATGTA 600

EF428204.1 ............................................................ 444

c79321_g3_i1 ............................................................ 600

Consensus TGTCGGATCGGCAGTGCTAAGGTGAGTGGACAGCCCCTGGGGGAAGCCCTAGTGGTGGGA 660

EF428204.1 ............................................................ 504

c79321_g3_i1 ............................................................ 660

Consensus GGAGAATCGCTGCTTGAAGCACCCTTCGACGGCATCCTGGGTCTGGCTTACCCGAGCATT 720

EF428204.1 ............................................................ 564

c79321_g3_i1 ............................................................ 720

Consensus GCCGTGGATGGAGTGGTGCCCGTCTTCGACAACATGATGAAGCAGGGTCTGCTTGGGGAG 780

EF428204.1 ............................................................ 624

c79321_g3_i1 ............................................................ 780

Consensus CAGAATGTCTTCTCCGTCTACCTGAACAGGGACCCCTCCAGCAAGGAGGGCGGGGAGGTC 840

EF428204.1 ............................................................ 684

c79321_g3_i1 ............................................................ 840

Consensus CTGTTCGGCGGCATCGACCACGATCACTACAAGGGCAGCATCACCTACGTCCCCGTCACG 900

EF428204.1 ............................................................ 744

c79321_g3_i1 ............................................................ 900

Consensus GCCAAGGGCTACTGGCAGTTCCACGTGGACGGTGTCAAGTCGGTTAGCGCCTCAAAATCG 960

EF428204.1 ............................................................ 804

c79321_g3_i1 ............................................................ 960

Consensus GCACCGGAGTTGCTGTGCAAGGATGGTTGCGAAGCCATTGCGGACACGGGCACCTCGCTC 1020

EF428204.1 ............................................................ 864

c79321_g3_i1 ............................................................ 1020

Consensus ATCACCGGACCACCCGAAGAGGTGGACTCCCTGAACCAGTACCTCGGAGGCACTAAGACA 1080

EF428204.1 ............................................................ 924

c79321_g3_i1 ............................................................ 1080

Consensus GAAGGCGGACAGTACCTACTCGACTGTGACAAGCTGGAGAGCCTACCGAATGTCACCTTC 1140

EF428204.1 ............................................................ 984

c79321_g3_i1 ............................................................ 1140

Consensus ACAATTTCCGGWAAAGAATTCTCGCTCCGCAGCAAGGACTACGTCCTGAAGGTAAATCAA 1200

EF428204.1 ...........A................................................ 1044

c79321_g3_i1 ...........T................................................ 1200

Consensus CAAGGTCAAACGCTTTGCGTGAGCGGTTTCATGRGCCTGGAGATGCCTCAACCCCTGTGG 1260

EF428204.1 .................................A.......................... 1104

c79321_g3_i1 .................................G.......................... 1260

Consensus ATTYTCGGAGACGTGTTCCTGGGCCCTTACTACMCCATCTTCGACCGGGACCAGGATCGG 1320

EF428204.1 ...T.............................C.......................... 1164

c79321_g3_i1 ...C.............................A.......................... 1320

Consensus GTCGGCTTCGCGGAAGTTGCCTAGGCCGTAASATYTCCGACTTCKTCCGCTGCAGAGCCT 1380

EF428204.1 ...............................G..T.........G............... 1224

c79321_g3_i1 ...............................C..C.........T............... 1380

Consensus TTTCAGKCAACGGKGGATCGCACTAGAAAAWCCTGGGTTTCCMCCTGATCATCTTGACTG 1440

EF428204.1 ......T......G................A...........C................. 1284

c79321_g3_i1 ......G......T................T...........A................. 1440

Consensus GTTGTTTTCGCCGTTATTTGAAAAAAAATAAAAATAAATAAAGCGATAAAATAATAAAAT 1500

EF428204.1 .........................----------------------------------- 1344

c79321_g3_i1 ............................................................ 1500

Consensus GCCGAGAAAATACTTTCTTTTTTCTTTTGTAAAAAAA 1537

EF428204.1 ------------------------------------- 1309

c79321_g3_i1 ..................................... 1537
