# Supplementary material for: Catalogue of stage-specific transcripts in Ixodes ricinus and their potential functions during the tick life-cycle
Source: Parasit Vectors. 2020 Jun 16;13:311. doi: 10.1186/s13071-020-04173-4 (PMC7296661; doi:10.1186/s13071-020-04173-4)
Supplement: Supplementary file 5 — Additional file 5: Alignment S2. Alignment of cathepsin D2 (GenBank: HQ615697.1) query sequence and a corresponding transcript recovered from Ixodes ricinus stage-specific transcriptome assembly (c81800_g1_i2). [file 13071_2020_4173_MOESM5_ESM.docx]

**Additional file 5: Alignment S2.** Alignment of cathepsin D2 (GenBank: HQ615697.1) query sequence and a corresponding transcript recovered from *Ixodes ricinus* stage-specific transcriptome assembly (c81800_g1_i2). Dots indicate agreements, hashes an absence of sequence in the alignment. Underlined sequence in the Consensus represent an ORF.

Consensus GCACACAATCTCCCGGGGACGTGATATACAGCGCTCATGTTGCAGCCGGCGCGGGACAGA 60

HQ615697.1 ------------------------------------------------------------

c81800_g1_i2 ............................................................ 60

Consensus GGACAGGGTCCGATAGGCATCAGCGATACGCTTATCCAGGACGCCGGGGAGCGCGAGTCG 120

HQ615697.1 ------------------------------------------------------------

c81800_g1_i2 ............................................................ 120

Consensus AATCATWCTCGAGTGCTCACCCGCTCCGCCGTCGCTGTCACATCTCCTGTAACATGGACG 180

HQ615697.1 ----..A..................................................... 56

c81800_g1_i2 ......T..................................................... 180

Consensus GCAAGCTGGCGCTCGTGGTTGCGCTCCTATCGCTATTTGGAGGCGCCRTAGGGGTCCTCA 240

HQ615697.1 ...............................................A............ 116

c81800_g1_i2 ...............................................G............ 240

Consensus GGATGCCCYTGCACAAGATGCAGAGTGCCCGGGCGCACCTGCTCGACGCAACCACCCCCC 300

HQ615697.1 ........C................................................... 176

c81800_g1_i2 ........T................................................... 300

Consensus TGACTCGACCGGCGGTGCACGSAACTCGTGGCCCCATCCCGGAGCCGCTCAAGAACTACT 360

HQ615697.1 .....................C...................................... 236

c81800_g1_i2 .....................G...................................... 360

Consensus TGGACGCCCAGTACTACGGCGAGATCACGCTGGGCACTCCCCCACAAAGCTTCCGGGTYG 420

HQ615697.1 ..........................................................C. 296

c81800_g1_i2 ..........................................................T. 420

Consensus TGTTTGACACTGGATCATCCAACTTGTGGGTGCCTTCTGCCAAGTGCCCTTTCACCAACA 480

HQ615697.1 ............................................................ 356

c81800_g1_i2 ............................................................ 480

Consensus TTGCCTGCCTGCTGCATCGCAAGTACTACAGCCGCAAGTCGAGCACGTACGTGAAGAACG 540

HQ615697.1 ............................................................ 416

c81800_g1_i2 ............................................................ 540

Consensus GCACCCAGTTCGAGATTCGCTACGGSAGCGGGAGTGTGCGGGGCGAGCTGAGCACGGACA 600

HQ615697.1 .........................G.................................. 476

c81800_g1_i2 .........................C.................................. 600

Consensus CGATGGGCGTCGGCGACAGCAGCGTGACGGGTCAGACGTTCGCCGAGATCCTGCACGAGT 660

HQ615697.1 ............................................................ 536

c81800_g1_i2 ............................................................ 660

Consensus CCGGCCTGGCCTTCCTGGCGGCCAAGTTCGACGGCATCCTCGGCCTGGGCTACCCTGAGA 720

HQ615697.1 ............................................................ 596

c81800_g1_i2 ............................................................ 720

Consensus TCTCGGTGCTGGGCGTGCCGACCGTCTTCGACACGATGGTGGCTCAGGGCGTGGCTGCCA 780

HQ615697.1 ............................................................ 656

c81800_g1_i2 ............................................................ 780

Consensus AGCCCGTCTTCTCCGTCTTCCTGGACCGCAACGCAAGCGACCCCGCTGGGGGAGAGGTGC 840

HQ615697.1 ............................................................ 716

c81800_g1_i2 ............................................................ 840

Consensus TCTTCGGCGGCATCGACGAATCCCACTACACCGGGAACATCAGCTACGTGCCGGTCTCCA 900

HQ615697.1 ............................................................ 776

c81800_g1_i2 ............................................................ 900

Consensus AGCGGGGCTACTGGCAGGTCCACATGGACGGGACTCGGGTGGGAAATAACGGCAGCTTCT 960

HQ615697.1 ............................................................ 836

c81800_g1_i2 ............................................................ 960

Consensus GCAGCGGCGGCTGCGAAGCTATCCTTGACACTGGAACCTCGTTGATCGCGGGTCCCTCGG 1020

HQ615697.1 ............................................................ 896

c81800_g1_i2 ............................................................ 1020

Consensus ACGAAATTGAGAAGCTCAACTTGCTCATCGGAGCTGCGCCATTTGCTTCTGGAGAGTACA 1080

HQ615697.1 ............................................................ 956

c81800_g1_i2 ............................................................ 1080

Consensus TCGTGAGCTGCAAGAGCATCGACAAGCTGCCCAAGATCACCTTCACCCTCGCGGGCAAGG 1140

HQ615697.1 ............................................................ 1016

c81800_g1_i2 ............................................................ 1140

Consensus ACTTCGTGCTCGAAGGGAAGGACTACGTCTTGCAGATGAGCTCTGCAGGAGTGCCTCTCT 1200

HQ615697.1 ............................................................ 1076

c81800_g1_i2 ............................................................ 1200

Consensus GCCTCAGCGGCTTCATYGGCCTGGACGTCCCTGCTCCGTTGGGGCCTCTCTGGATCCTGG 1260

HQ615697.1 ................T........................................... 1136

c81800_g1_i2 ................C........................................... 1260

Consensus GCGACGTGTTCATCGGTCGCTATTACACCATCTTCGACCGGGGAAACGACCGCGTGGGAC 1320

HQ615697.1 ............................................................ 1196

c81800_g1_i2 ............................................................ 1320

Consensus TCGCRAACGCTCGCTGAGACGCTGCGAATGCTGAAGACAGCCAGAACCACTTGTGGGGGG 1380

HQ615697.1 ....G....................................................... 1256

c81800_g1_i2 ....A....................................................... 1380

Consensus CCCCCGAAATTTACTCCCCGCCTGTGTTYGTCTAATGCGATGATTCACCCTAATCACAGC 1440

HQ615697.1 ............................C............................... 1316

c81800_g1_i2 ............................T............................... 1440

Consensus GTACTAGAATAGRGGGTAGTGTTTGGACTGGCGCGACTCAACAATGGCAACTTGTGAGGC 1500

HQ615697.1 ............G............................................... 1376

c81800_g1_i2 ............A............................................... 1500

Consensus TGCGCGTGTAGACAACAATCGCGGCGCTGTAGTCAACAGGGTAGCCGAAAGTCGCGCCTG 1560

HQ615697.1 ............................................................ 1436

c81800_g1_i2 ............................................................ 1560

Consensus CAGTCCGCACGTGCRCGTGCACGCGGTGCACGCGCACAACCACCACCATACTGACCCTAT 1620

HQ615697.1 ..............G............................................. 1496

c81800_g1_i2 ..............A............................................. 1620

Consensus CGCCAATGCGCCRRTGTCGACACGTATCGCGATCTACACCACACGCCTCACGCGAGCCGC 1680

HQ615697.1 ............AA.............................................. 1556

c81800_g1_i2 ............GG.............................................. 1680

Consensus TCCGGTCCCAACACTACCCCGTATTCTAGTACACTGTACCCAAATCACGGGGCACCAATT 1740

HQ615697.1 ............................................................ 1616

c81800_g1_i2 ............................................................ 1740

Consensus TGCGCGAGAGGATCCCTGTCTTCACCTCCTTCCGYTTTCTGTTTATAAGAGAAAAGACGA 1800

HQ615697.1 ..................................C......................... 1676

c81800_g1_i2 ..................................T......................... 1800

Consensus TTATTAAATAAAATATGTGAAAAGGKSACCCGGTTTTCGGAATGCGTGAAAGTTGGTACA 1860

HQ615697.1 .........................GC................................. 1736

c81800_g1_i2 .........................TG................................. 1860

Consensus CAGATGACAAATASAAGTCGYGTCTATGWTCGAGCRATAA 1900

HQ615697.1 .............C......C.......A......A.... 1776

c81800_g1_i2 .............G......T.......T......G---- 1896
