# Supplementary material for: Catalogue of stage-specific transcripts in Ixodes ricinus and their potential functions during the tick life-cycle
Source: Parasit Vectors. 2020 Jun 16;13:311. doi: 10.1186/s13071-020-04173-4 (PMC7296661; doi:10.1186/s13071-020-04173-4)
Supplement: Supplementary file 6 — Additional file 6: Alignment S3. Alignment of cathepsin D3 (GenBank: HQ615698.1) query sequence and a corresponding transcript recovered from Ixodes ricinus stage-specific transcriptome assembly (c81927_g1_i1). [file 13071_2020_4173_MOESM6_ESM.docx]

**Additional file 6: Alignment S3.** Alignment of cathepsin D3 (GenBank: HQ615698.1) query sequence and a corresponding transcript recovered from *Ixodes ricinus* stage specific transcriptome assembly (c81800_g1_i2). Dots indicate agreements, hashes an absence of sequence in the alignment. Underlined sequence in the Consensus represent an ORF.

Consensus TTGTTCAGAAACGAAACCAGGKCGSTTCTGATAAAGCTATCGAGGTCAAGTTCACAGTTY 60

HQ615698.1 .....................G..G..................................T 60

c81927_g1_i1 -------------------..T..C..................................C 41

Consensus GCAGAGACAGCTGAAAAAGCATCTTCCCAGGCGGGRCCTCATTTTSTCGCCGAAATGGGC 120

HQ615698.1 ...................................A.........C.............. 120

c81927_g1_i1 ...................................G.........G.............. 101

Consensus ARGAYGGACAAGTCGGCGGTCCTGGTCGTCTTCCTGGCTGCCTTGTCGGCCGTCAACTGC 180

HQ615698.1 .G..C....................................................... 180

c81927_g1_i1 .A..T....................................................... 161

Consensus CTGCTCAGTGTCCCCCTGCACCGCTTCAAGTCCGTGCGGCGTGCCCTGGAGGAGGTGGGC 240

HQ615698.1 ............................................................ 240

c81927_g1_i1 ............................................................ 221

Consensus ACGGAGGTGGTGGTGGCCCAGCCCAAGTACTACAACGAAGTGGGCGGCACCCTGCCCATT 300

HQ615698.1 ............................................................ 300

c81927_g1_i1 ............................................................ 281

Consensus CCGGAGCCCCTGTCCAACTACCTGGACGCCCAATACTATGGACCAATCAGCATCGGGAGC 360

HQ615698.1 ............................................................ 360

c81927_g1_i1 ............................................................ 341

Consensus CCTCCCCAGCCCTTYCGCGTGGTGTTTGACACGGGTTCCTCCAATCTCTGGGTGCCTTCT 420

HQ615698.1 ..............T............................................. 420

c81927_g1_i1 ..............C............................................. 401

Consensus AAGCAGTGCAAGTGGACCAACATCGCCTGCCTGCTCCACAAGAAGTATGACCACACACGC 480

HQ615698.1 ............................................................ 480

c81927_g1_i1 ............................................................ 461

Consensus TCCAGGAGCTACCGCAAGAATGGGACAGCCATTTCTCTGCGCTACGGCACGGGCAGCATG 540

HQ615698.1 ............................................................ 540

c81927_g1_i1 ............................................................ 521

Consensus ACTGGCTTCCTCAGCGTCGACACCGTGTCCCTGGCCGGCATCGAYGTYCACAATCAGACG 600

HQ615698.1 ............................................T..T............ 600

c81927_g1_i1 ............................................C..C............ 581

Consensus TTTGCRGAGGCCGTGACGGAGCCGGGCCTGACGTTTGTGGCTGCCAAGTTCGACGGCATC 660

HQ615698.1 .....A...................................................... 660

c81927_g1_i1 .....G...................................................... 641

Consensus TTGGGCCTGGGCTTCAGCAACATTGCAGTCATGGGAGCCCCCACGGTCTTYGACAACATG 720

HQ615698.1 ..................................................T......... 720

c81927_g1_i1 ..................................................C......... 701

Consensus GTGGCGCAGCTACTCGTGCCCAGGCCCGTCTTCTCCTTCTTCCTCAACCGGAACACGACT 780

HQ615698.1 ............................................................ 780

c81927_g1_i1 ............................................................ 761

Consensus TCCCCGACCGGTGGAGAGATCACTTTTGGCGGCACGGACGACCGCTTCTACTCTGGGGAC 840

HQ615698.1 ............................................................ 840

c81927_g1_i1 ............................................................ 821

Consensus ATCAGCTACGTCCCTGTCTCAACCAAAGGCTACTGGCAGTTCACGGTGGACAACATTGTG 900

HQ615698.1 ............................................................ 900

c81927_g1_i1 ............................................................ 881

Consensus GTGAAGAACAGCTCGTTCAAGCTGTGTGCGGAGGGCTGCGAGGCCATCGCGGACACGGGC 960

HQ615698.1 ............................................................ 960

c81927_g1_i1 ............................................................ 941

Consensus ACCTCCCTCATGGCGGGGCCTTCCCTGGAGATCATGAAGCTGCAGAAGCTCATCGGGGCA 1020

HQ615698.1 ............................................................ 1020

c81927_g1_i1 ............................................................ 1001

Consensus CTGCCTTTCTCCCACGGGCAGTACACCGTGCGCTGCGAAGACATTCACAAGCTGCCGGAC 1080

HQ615698.1 ............................................................ 1080

c81927_g1_i1 ............................................................ 1061

Consensus ATCAAGTTCCACATTGGCGGGCAGGAGTACGTGCTCACCGGCAGCGACTACGTCCTCAAG 1140

HQ615698.1 ............................................................ 1140

c81927_g1_i1 ............................................................ 1121

Consensus ATCACGCAGTTTGGCCGGATGATCTGCCTGTCCGGCTTTGTGGGGCTGGACATTCCGGAG 1200

HQ615698.1 ............................................................ 1200

c81927_g1_i1 ............................................................ 1181

Consensus CCCCGCGGACCRCTCTGGATCCTGGGGGACGTGTTCATCGGCCGCTACTACACCGTCTTC 1260

HQ615698.1 ...........A................................................ 1260

c81927_g1_i1 ...........G................................................ 1241

Consensus GACTACGGGGCCTCCAGGGTGGGCTTCGCCAAGGCCAGGGAGGTCTACTGAGTGGCCCTG 1320

HQ615698.1 ............................................................ 1320

c81927_g1_i1 ............................................................ 1301

Consensus GTCTACTGAGTGTCCCTCCATTTCGAAGGAGCGTGCCCGAGACGGGGTACTGGTGTGGAC 1380

HQ615698.1 ............................................................ 1380

c81927_g1_i1 ............................................................ 1361

Consensus TGATTGTTGTGTCTGTTCACACCTGCTCGTTCTTTTCTGCCCGGCGACGCAAAAACACAA 1440

HQ615698.1 ............................................................ 1440

c81927_g1_i1 ............................................................ 1421

Consensus GTGACGTTCTTGWCRWCCGCGTTTTCGTGTCGTTGGCCTCGTGCCTGCTGCACTAGTGGT 1500

HQ615698.1 ............A.AA-------------------------------------------- 1500

c81927_g1_i1 ............T.GT............................................ 1481

Consensus AACTTTAGCGCAACCCCACGCTTTACACGTCGAGTCTTCGTAAAATTGAGCGATGTGGGG 1560

HQ615698.1 ------------------------------------------------------------ 1560

c81927_g1_i1 ............................................................ 1541

Consensus GTTTCTAAGCAAGTTTCTAACTAGTTTAGAAGCTTGAACAATTATTTTCACTAATTAACC 1620

HQ615698.1 ------------------------------------------------------------ 1620

c81927_g1_i1 ............................................................ 1601

Consensus CTTTGACCATCATCACCGCACGTATATGGCAGGGATTACACGTTTGGAAGTGCCCGCCTA 1680

HQ615698.1 ------------------------------------------------------------ 1680

c81927_g1_i1 ............................................................ 1661

Consensus AACACGAAACCACGCGCTTTGCGGAAGGTGTTTCTATTTGTTGTCTTCT 1729

HQ615698.1 ------------------------------------------------- 1456

c81927_g1_i1 ................................................. 1710
