# Supplementary material for: Catalogue of stage-specific transcripts in Ixodes ricinus and their potential functions during the tick life-cycle
Source: Parasit Vectors. 2020 Jun 16;13:311. doi: 10.1186/s13071-020-04173-4 (PMC7296661; doi:10.1186/s13071-020-04173-4)
Supplement: Supplementary file 7 — Additional file 7: Alignment S4. Alignment of Iris (GenBank: AJ269658.2) query sequence and four corresponding transcripts representing Trinity assembler isoforms recovered from Ixodes ricinus stage-specific transcriptome assembly (c83951_g1_i2, c83951_g1_i5, c83951_g1_i3, c83951_g1_i1). [file 13071_2020_4173_MOESM7_ESM.docx]

**Additional file 7: Alignment S4.** Alignment of Iris (GenBank: AJ269658.2) query sequence and four corresponding transcripts representing Trinity assembler isoforms recovered from *Ixodes ricinus* stage-specific transcriptome assembly (c83951_g1_i2, c83951_g1_i5, c83951_g1_i3, c83951_g1_i1). Dots indicate agreements, hashes an absence of sequence in the alignment. Underlined sequence in the Consensus represent an ORF.

The four transcripts (c83951_g1_i2, c83951_g1_i5, c83951_g1_i3, c83951_g1_i1) represent “Trinity isoforms” produced by Trinity assembler and do not necessarily correspond to actual transcript isoforms.

Consensus GGGACAGGCTGCTGTACGCCTGGGGCCGTGCAAGCGAAGGCAGCGAAGGCTGCGAGTGTA 60

AJ269658.2 ------------------------------------------------------------

c83951_g1_i2 -----------------........................................... 43

c83951_g1_i5 -----------------........................................... 43

c83951_g1_i3 -----------------........................................... 43

c83951_g1_i1 .................ATG.A.T.......T....G..C.T...G.............. 60

Consensus CGTGCAGTTCGGAAGTGCAATATCCTGTTATTAAGCTCTAATTAGCACACTGTGAGTCGA 120

AJ269658.2 ------------------------------------------------------------

c83951_g1_i2 ............................................................ 103

c83951_g1_i5 ............................................................ 103

c83951_g1_i3 ............................................................ 103

c83951_g1_i1 ..A...T.....................C....G..A..G..ATT..T..C......T.. 120

Consensus TCAGAGGCCTCTCTTAACGCC--------------------------------------- 141

AJ269658.2 ------------------------------------------------------------

c83951_g1_i2 .....................--------------------------------------- 124

c83951_g1_i5 .....................--------------------------------------- 124

c83951_g1_i3 .....................--------------------------------------- 124

c83951_g1_i1 ...................GTGAATATACGTGTTTGTAGAACTCTTAACAACTCGTGACA 180

Consensus ------------------------------------------------------------ 141

AJ269658.2 ------------------------------------------------------------

c83951_g1_i2 ------------------------------------------------------------ 124

c83951_g1_i5 ------------------------------------------------------------ 124

c83951_g1_i3 ------------------------------------------------------------ 124

c83951_g1_i1 GCGTCGGTAATTGGAGACGACGATTTGTTTACACGAACGGCTTGGATGACGACGCCCACC 240

Consensus ---------------------------------------ACATTGAAAAAGGATCCAAGA 162

AJ269658.2 -----------------------------------------------------------. 1

c83951_g1_i2 ---------------------------------------..................... 145

c83951_g1_i5 ---------------------------------------..................... 145

c83951_g1_i3 ---------------------------------------..................... 145

c83951_g1_i1 TTGCGAAGGACGTAGCAATCGCCGCCAAAGCTTTCAGAA................T.... 300

Consensus TGGAGGCAAGTCTGAGCAACCACATCCTTAACTTCTCCGTCGACCTATACAAGCAGCTGA 222

AJ269658.2 ..............................................C.......G..... 61

c83951_g1_i2 ............................................................ 205

c83951_g1_i5 ............................................................ 205

c83951_g1_i3 ............................................................ 205

c83951_g1_i1 ................................................T........... 360

Consensus AACCCTCCGGCAAAGACACGGCAGGAAACGTCTTCTGCTCACCATTCAGTATTGCAGCTG 282

AJ269658.2 ............................................................ 121

c83951_g1_i2 ............................................................ 265

c83951_g1_i5 ............................................................ 265

c83951_g1_i3 ............................................................ 265

c83951_g1_i1 ....A....................................................... 420

Consensus CACTGTCCATGGCCCTCGCAGGAGCTAGAGGCAACACTGCCAAGCAAATCGCTGCCATCC 342

AJ269658.2 ............................................................ 181

c83951_g1_i2 ............................................................ 325

c83951_g1_i5 ............................................................ 325

c83951_g1_i3 ............................................................ 325

c83951_g1_i1 ............................................................ 480

Consensus TGCACTCAAACGACGACAAGATCCACGACCACTTCTCCAACTTCCTTTGCAAGCTACCCA 402

AJ269658.2 .....................................G.G.................... 241

c83951_g1_i2 ............................................................ 385

c83951_g1_i5 ............................................................ 385

c83951_g1_i3 ............................................................ 385

c83951_g1_i1 ............................................................ 540

Consensus GTTACGCCCCAGATGTGGCCCTGCACATCGCCAATCGCATGTACTCTGAGCAGACCTTCC 462

AJ269658.2 ............................................................ 301

c83951_g1_i2 ............................................................ 445

c83951_g1_i5 ............................................................ 445

c83951_g1_i3 ............................................................ 445

c83951_g1_i1 ............................................................ 600

Consensus ATCCGAAAGCGGAGTACACAACCCTGTTGCAGAAGTCCTACGACAGCACCATCAAGGCTG 522

AJ269658.2 ...............................A............................ 361

c83951_g1_i2 ............................................................ 505

c83951_g1_i5 ............................................................ 505

c83951_g1_i3 ............................................................ 505

c83951_g1_i1 ............................................................ 660

Consensus TTGACTTTGCAGGAAATGCCGACAGGGTCCGTCTGGAGGTCAATGCCTGGGTTGAGGAAG 582

AJ269658.2 ............................................................ 421

c83951_g1_i2 ............................................................ 565

c83951_g1_i5 ............................................................ 565

c83951_g1_i3 ............................................................ 565

c83951_g1_i1 ............................................................ 720

Consensus TCACCAGGTCAAAGATCAGGGACCTGCTCGCACCTGGAACTGTTGATTCATCGACATCAC 642

AJ269658.2 ............................................................ 481

c83951_g1_i2 ............................................................ 625

c83951_g1_i5 ............................................................ 625

c83951_g1_i3 ............................................................ 625

c83951_g1_i1 ............................................................ 780

Consensus TTATATTGGTGAATGCCATTTACTTCAAAGGTCTGTGGGACTCTCAGTTCAAGCCTAGTG 702

AJ269658.2 ............................................................ 541

c83951_g1_i2 ............................................................ 685

c83951_g1_i5 ............................................................ 685

c83951_g1_i3 ............................................................ 685

c83951_g1_i1 ............................................................ 840

Consensus CTACGAAGCCGGGAGATTTTCACTTGACACCACAGACCTCAAAGAAAGTGGACATGATGC 762

AJ269658.2 ............................................................ 601

c83951_g1_i2 ............................................................ 745

c83951_g1_i5 ............................................................ 745

c83951_g1_i3 ............................................................ 745

c83951_g1_i1 ............................................................ 900

Consensus ACCAGAAAGGAGACTTCAAGATGGGTCACTGCAGCGACCTCAAGGTCACTGCGCTTGAGA 822

AJ269658.2 ............................................................ 661

c83951_g1_i2 ............................................................ 805

c83951_g1_i5 ............................................................ 805

c83951_g1_i3 ............................................................ 805

c83951_g1_i1 ............................................................ 960

Consensus TACCCTACAAAGGCAACAAGACGTCGATGGTCATTCTCCTGCCCGAAGATGTGGAGGGAC 882

AJ269658.2 ............................................................ 721

c83951_g1_i2 ............................................................ 865

c83951_g1_i5 ............................................................ 865

c83951_g1_i3 ............................................................ 865

c83951_g1_i1 ............................................................ 1020

Consensus TCTCAGTCCTGGAGGAACACTTGACCGCTCCGAAACTGTCGGCTCTGCTCAGCGGCATGT 942

AJ269658.2 ..................................................G......... 781

c83951_g1_i2 ............................................................ 925

c83951_g1_i5 ............................................................ 925

c83951_g1_i3 ............................................................ 925

c83951_g1_i1 ..................................................G......... 1080

Consensus ATAGGAAATCCGATGTCAACTTGCGCTTGCCGAAGTTCAAACTGGAGCAGTCCATAGGTT 1002

AJ269658.2 ..GT..CG.................................................... 841

c83951_g1_i2 ............................................................ 985

c83951_g1_i5 ............................................................ 985

c83951_g1_i3 ............................................................ 985

c83951_g1_i1 ..GT..CG.................................................... 1140

Consensus TGAAGGATGTACTGATGGCGATGGGAGTCAAGGATTTTTTCACGTCCCTTGCGGATCTTT 1062

AJ269658.2 .....................................C.....A........A....... 901

c83951_g1_i2 ............................................................ 1045

c83951_g1_i5 ............................................................ 1045

c83951_g1_i3 ............................................................ 1045

c83951_g1_i1 .....................................----------------------- 1200

Consensus CTGGCATCAGCGCTACGGGGAATCTTTGCGCTTCGGATGTCATCCACAAGGCTTTTGTGG 1122

AJ269658.2 ..............G..........G............A..................... 961

c83951_g1_i2 ............................................................ 1105

c83951_g1_i5 ............................................................ 1105

c83951_g1_i3 ............................................................ 1105

c83951_g1_i1 ------------------------------------------------------------ 1260

Consensus AAGTTAATGAGGAGGGCACAGAGGCTGCAGCTGCCACTGCCATACCCATTATGTTGATGT 1182

AJ269658.2 ............................................................ 1021

c83951_g1_i2 ............................................................ 1165

c83951_g1_i5 ............................................................ 1165

c83951_g1_i3 ............................................................ 1165

c83951_g1_i1 ------------------------------------------------------------ 1320

Consensus GTGCGAGATTTCCACAGGTGGTGAACTTTTTCGTTGACCGCCCATTCATGTTCTTGATCC 1242

AJ269658.2 .Y.......................................................... 1081

c83951_g1_i2 ............................................................ 1225

c83951_g1_i5 ............................................................ 1225

c83951_g1_i3 ............................................................ 1225

c83951_g1_i1 ------------------------------------------------------------ 1380

Consensus ACAGCCATGATCCAGATGTTGTTCTCTTCATGGGATCCATCCGTGAGCTCTAAAAAGCAT 1302

AJ269658.2 .....................................................------- 1141

c83951_g1_i2 ............................................................ 1285

c83951_g1_i5 ............................................................ 1285

c83951_g1_i3 ............................................................ 1285

c83951_g1_i1 ------------------------------------------------------------ 1440

Consensus ATTCTTAACGGCGGCCAATCAGTCTGTGGGGTTATCTCTTAGTCACTAATGTGTAACAAT 1362

AJ269658.2 ------------------------------------------------------------ 1201

c83951_g1_i2 ............................................................ 1345

c83951_g1_i5 ............................................................ 1345

c83951_g1_i3 ............................................................ 1345

c83951_g1_i1 ------------------------------------------------------------ 1500

Consensus TCTGCAATATTCAGCTTGTGTATTTCAGTAACTTGCTAGATCTTTGTGTTGTTGATGTTA 1422

AJ269658.2 ------------------------------------------------------------ 1261

c83951_g1_i2 ............................................................ 1405

c83951_g1_i5 ............................................................ 1405

c83951_g1_i3 ............................................................ 1405

c83951_g1_i1 ------------------------------------------------------------ 1560

Consensus GGCTTCTTGTGAGATATGTTGTGCATTAAAATAGAGATGAGGACTAAAGTAAATGAACTA 1482

AJ269658.2 ------------------------------------------------------------ 1321

c83951_g1_i2 ............................................................ 1465

c83951_g1_i5 ............................................................ 1465

c83951_g1_i3 ............................................................ 1465

c83951_g1_i1 ------------------------------------------------------------ 1620

Consensus AGTTAAAGCCATGAAACAGTCACCAACAAGCATTGTGAGCTGGTGAAGAAGGCACAGTAA 1542

AJ269658.2 ------------------------------------------------------------ 1381

c83951_g1_i2 ............................................................ 1525

c83951_g1_i5 ............................................................ 1525

c83951_g1_i3 ............................................................ 1525

c83951_g1_i1 ------------------------------------------------------------ 1680

Consensus AATATGAAGATGAACGTAAAAGTACCTAGCAACATTTCAGTTTTTATATATTGTTTATTA 1602

AJ269658.2 ------------------------------------------------------------ 1441

c83951_g1_i2 ............................................................ 1585

c83951_g1_i5 ............................................................ 1585

c83951_g1_i3 ............................................................ 1585

c83951_g1_i1 ------------------------------------------------------------ 1740

Consensus TTCTAGCATCCTTGCTGCAAATTGAACTTGTCATGCAGAAAGCCCTTACTTTTGCTGAGC 1662

AJ269658.2 ------------------------------------------------------------ 1501

c83951_g1_i2 ............................................................ 1645

c83951_g1_i5 ............................................................ 1645

c83951_g1_i3 ............................................................ 1645

c83951_g1_i1 ------------------------------------------------------------ 1800

Consensus TGTTTCCATAGAATTATGTGCTCAAATAACTAAATGAGTGTGAACTAGCTGATGAGCTAG 1722

AJ269658.2 ------------------------------------------------------------ 1561

c83951_g1_i2 ............................................................ 1705

c83951_g1_i5 ............................................................ 1705

c83951_g1_i3 ............................................................ 1705

c83951_g1_i1 ------------------------------------------------------------ 1860

Consensus TTAAATTAATGTGAACAACCTGATGTCTTGTGTTCATAGAGTCGATTTGCATGAAAACAT 1782

AJ269658.2 ------------------------------------------------------------ 1621

c83951_g1_i2 ............................................................ 1765

c83951_g1_i5 ............................................................ 1765

c83951_g1_i3 ...........................................A...........T.T.. 1765

c83951_g1_i1 ------------------------------------------------------------ 1920

Consensus GTGTTATGTAAAGTTCACCGAAATTGTCTGACACGGGGCGACCATTTGAAGAAATATTTG 1842

AJ269658.2 ------------------------------------------------------------ 1681

c83951_g1_i2 ............................................................ 1825

c83951_g1_i5 ............................................................ 1825

c83951_g1_i3 ...----------------------------...AA......................G. 1797

c83951_g1_i1 ------------------------------------------------------------ 1980

Consensus AATAAGTTTGTGCAAATTTCTATAAATCCTTGGTTGTGGCAGCACGGGGCAAGTTTGCCT 1902

AJ269658.2 ------------------------------------------------------------ 1741

c83951_g1_i2 ............................................................ 1885

c83951_g1_i5 ............................................................ 1885

c83951_g1_i3 ..............................................AA....A....... 1857

c83951_g1_i1 ------------------------------------------------------------ 2040

Consensus ACAAAAAAAGAAAAAAAGCAACACAAACATAGATTGT----------------------- 1939

AJ269658.2 ------------------------------------------------------------ 1801

c83951_g1_i2 .....................................----------------------- 1922

c83951_g1_i5 .....................................----------------------- 1922

c83951_g1_i3 ......----.........T.......T...C.....ACAGTCTGCTTTGAGCCACCTAA 1913

c83951_g1_i1 ------------------------------------------------------------ 2100

Consensus ----------------ACCTTTTTATGTTATGAGACAGCAAG-CTTTAACAAAAATGCTA 1982

AJ269658.2 ------------------------------------------------------------ 1861

c83951_g1_i2 ----------------..........................-................. 1965

c83951_g1_i5 ----------------..........................-................. 1965

c83951_g1_i3 ACTTTCATATACAGGG........................G.TT.....A......T... 1973

c83951_g1_i1 ------------------------------------------------------------ 2160

Consensus TTTGCATGATCACTGAGTACTTGTTGGGCTTGTGCAGTATAGTACATAATCAAAGTAGTG 2042

AJ269658.2 ------------------------------------------------------------ 1921

c83951_g1_i2 ............................................................ 2025

c83951_g1_i5 ............................................................ 2025

c83951_g1_i3 ............................................................ 2033

c83951_g1_i1 ------------------------------------------------------------ 2220

Consensus CACTTTGTATATCCGTAACAAAATGCAGTACTGTGATGTCCTGCTACCAGGACCAATTTT 2102

AJ269658.2 ------------------------------------------------------------ 1981

c83951_g1_i2 ............................................................ 2085

c83951_g1_i5 ............................................................ 2085

c83951_g1_i3 ............................................................ 2093

c83951_g1_i1 ------------------------------------------------------------ 2280

Consensus AAATATGATCGCTCGGATTCCTGAATCTTAGGATCTCTGCTCAATGAGACCGCGCATTTT 2162

AJ269658.2 ------------------------------------------------------------ 2041

c83951_g1_i2 ............................................................ 2145

c83951_g1_i5 ............................................................ 2145

c83951_g1_i3 ..........C...A............................................. 2153

c83951_g1_i1 ------------------------------------------------------------ 2340

Consensus TCTGAATCTTTATGCGAATATATAGTCATGATACCAAAGGTGCTATTTTAAACTGCTAAC 2222

AJ269658.2 ------------------------------------------------------------ 2101

c83951_g1_i2 ............................................................ 2205

c83951_g1_i5 ............................................................ 2205

c83951_g1_i3 ............................................................ 2213

c83951_g1_i1 ------------------------------------------------------------ 2400

Consensus GAGCAATATGTACCGGTGCATTTTTTTCTTCTTGCATTTCCATTTATTATTTTGATGCTA 2282

AJ269658.2 ------------------------------------------------------------ 2161

c83951_g1_i2 ............................................................ 2265

c83951_g1_i5 ............................................................ 2265

c83951_g1_i3 ............................................................ 2273

c83951_g1_i1 ------------------------------------------------------------ 2460

Consensus TTCCTATTTGCCGCAATTATATAGCACAAATGCACCTGCACAGATCTTTTGAGTTGGAAC 2342

AJ269658.2 ------------------------------------------------------------ 2221

c83951_g1_i2 ............................................................ 2325

c83951_g1_i5 ............................................................ 2325

c83951_g1_i3 ............................................................ 2333

c83951_g1_i1 ------------------------------------------------------------ 2520

Consensus ACTAATTTAGGGGCTGCTGTGTGTAGAACTAGAAACTGTTATGTTTTTATATTTTGGTGT 2402

AJ269658.2 ------------------------------------------------------------ 2281

c83951_g1_i2 ............................................................ 2385

c83951_g1_i5 ............................................................ 2385

c83951_g1_i3 ............................................................ 2393

c83951_g1_i1 ------------------------------------------------------------ 2580

Consensus AGGGTTCTGCTTTGACAACAGGAGATCCATAAGAAGCATGTCATGCAAAAGGATAAATAA 2462

AJ269658.2 ------------------------------------------------------------ 2341

c83951_g1_i2 ............................................................ 2445

c83951_g1_i5 ............................................................ 2445

c83951_g1_i3 ............................................................ 2453

c83951_g1_i1 ------------------------------------------------------------ 2640

Consensus ATGCGCAAATAGCAGTATCCGTGCACTTATATGGCCCTCAGTGGCTTCACAAAGGGAAGG 2522

AJ269658.2 ------------------------------------------------------------ 2401

c83951_g1_i2 ............................................................ 2505

c83951_g1_i5 ............................................................ 2505

c83951_g1_i3 ............................................................ 2513

c83951_g1_i1 ------------------------------------------------------------ 2700

Consensus GGGATGGGGGGCAGACCGTTCTGGGTGTAGCTGGGCAGTGAAAGGGGGTTGTTGTGGCAG 2582

AJ269658.2 ------------------------------------------------------------ 2461

c83951_g1_i2 ............................................................ 2565

c83951_g1_i5 ............................................................ 2565

c83951_g1_i3 ............................................................ 2573

c83951_g1_i1 ------------------------------------------------------------ 2760

Consensus CGGCCCGCCGCTCCGGGTGCCGCGGACCCTAGTGATGCCACTGATGGCSCKTACAGGCAA 2642

AJ269658.2 ------------------------------------------------------------ 2521

c83951_g1_i2 ................................................G.T......... 2625

c83951_g1_i5 ................C.C.G.AA------------------C.....------------ 2607

c83951_g1_i3 ................................................C.G..------- 2633

c83951_g1_i1 ------------------------------------------------------------ 2820

Consensus TGTTCAAGATTTGTTTT 2659

AJ269658.2 ----------------- 1134

c83951_g1_i2 ................. 2642

c83951_g1_i5 ----------------- 2595

c83951_g1_i3 ----------------- 2626

c83951_g1_i1 ----------------- 1177
