# Supplementary material for: Catalogue of stage-specific transcripts in Ixodes ricinus and their potential functions during the tick life-cycle
Source: Parasit Vectors. 2020 Jun 16;13:311. doi: 10.1186/s13071-020-04173-4 (PMC7296661; doi:10.1186/s13071-020-04173-4)
Supplement: Supplementary file 8 — Additional file 8: Alignment S5. Alignment of ixoderin A (GenBank: AY341424.1) query sequence and a corresponding transcript recovered from Ixodes ricinus stage-specific transcriptome assembly (c80994_g1_i1). [file 13071_2020_4173_MOESM8_ESM.docx]

**Additional file 8: Alignment S5.** Alignment of ixoderin A (GenBank: AY341424.1) query sequence and a corresponding transcript recovered from *Ixodes ricinus* stage-specific transcriptome assembly (c80994_g1_i1). Dots indicate agreements, hashes an absence of sequence in the alignment. Underlined sequence in the Consensus represent an ORF.

Consensus GCCACTTCCTTCGAGATCGCTCCATCATCTCGCGGTTTCAGTTGGCGGGGGAAAGACGGA 60

AY341424.1 ------------------------------------------------------------

c80994_g1_i1 ............................................................ 60

Consensus GCTTCGCCACCGACAGTCGCTCTGAACCTTGGCCGTCTGAGCCTTGCGTGACGTGATTGT 120

AY341424.1 ------------------------------------------------------------

c80994_g1_i1 ............................................................ 120

Consensus GTCAGTCCATAAAAGGCGAGCTACGAGACAGCCCATACACCAGCCGCCAGAAACGGCTAC 180

AY341424.1 ------------------------------------------------------------

c80994_g1_i1 ............................................................ 180

Consensus AGCAGACTTTCTTTTGTGCGGCTCCATCATGTCTTTTCAGAACGCTCGCCAGCATGACCG 240

AY341424.1 ------------------------------------------------------------

c80994_g1_i1 ............................................................ 240

Consensus CTCCGTCATCATGCTCCTGGGCATCCTCATCGGCATGGCGCCTGCCATCGTGGCGGTCTC 300

AY341424.1 ----------.................................................. 50

c80994_g1_i1 ............................................................ 300

Consensus GGCAAACCCTGTTGCGACCCTGGCAGTCCTAGAGAGAGCAGAGAATCACATMRGTGAACT 360

AY341424.1 ...................................................AA....... 110

c80994_g1_i1 ...................................................CG....... 360

Consensus GTCGAAGATCGTAKTGGACTTAAAGAAAAGCCTTCGCCCTCGAGATTGCGGAGACCTGCT 420

AY341424.1 .............G.............................................. 170

c80994_g1_i1 .............T.............................................. 420

Consensus AAAGGCTGGACAAATAAACAACGGAGTTTACGTCATTTTCCCGACCTCGGATTCCAAGGG 480

AY341424.1 ............................................................ 230

c80994_g1_i1 ............................................................ 480

Consensus AACRTCGGTCTATTGCGATATGAAGACCGACGGTGGAGGATGGACCGTTATTCAGAACAG 540

AY341424.1 ...A........................................................ 290

c80994_g1_i1 ...G........................................................ 540

Consensus AGGCCAGTATGGGAACAGTGTCTACTACTTCTACCGAAACTGGACGGAGTATGCAAATGG 600

AY341424.1 ............................................................ 350

c80994_g1_i1 ............................................................ 600

Consensus ATTTGGAGACAGGGATAAGGAATATTGGATTGGAAACGACGTGCTGCACACCCTTACATC 660

AY341424.1 ............................................................ 410

c80994_g1_i1 ............................................................ 660

Consensus GGAAGACAGGGGATTCTCTCTACGAATTGAACTGAAAAACGAGACAGGAGAAAGCCTKGT 720

AY341424.1 .........................................................G.. 470

c80994_g1_i1 .........................................................T.. 720

Consensus TGCAAACTACAAAATCTTCAAAGTCRSSKYYYMRGCTAACCTTTACAAAATGACGGTCGG 780

AY341424.1 .........................AGCGTCTCA.......................... 530

c80994_g1_i1 .........................GCGTCTCAG.......................... 780

Consensus YGGCTACTCCGGACCATCAGATTNCGGACTCGTTCAKCTMCACSAACGGAATCAACTTTA 840

AY341424.1 T......................C............T..C...C................ 590

c80994_g1_i1 C......................-............G..A...G................ 839

Consensus NCACCTTCGACAGKGACAACGATAATCACAACASCAACTGTGCTRCAACCTACAAAGGCG 900

AY341424.1 -............G...................G..........G............... 649

c80994_g1_i1 G............T...................C..........A............... 899

Consensus GYTGGTGGTACGCCGCCTGCCACAKCAGCAACCTCAATGGCCTTAACCTGAACGGGCCAC 960

AY341424.1 .C......................T................................... 709

c80994_g1_i1 .T......................G................................... 959

Consensus ACGCGAGCTTCGCTGACGGCATTGAGTGGTCCAGGCGGAACCACGTCGGAGGCTTGTACC 1020

AY341424.1 ............................................................ 769

c80994_g1_i1 ............................................................ 1019

Consensus ATTACTCCTATCCCGAAGCACGAATGATGATCCGGGAGGCCAACCCTATGCCCGAAGCAG 1080

AY341424.1 ............................................................ 829

c80994_g1_i1 ............................................................ 1079

Consensus TTTGACCTTGTCGGCGTGACCTTCCATGAAAGACTGAGAAAATGTGTATGAAATAATGTT 1140

AY341424.1 .....------------------------------------------------------- 889

c80994_g1_i1 ............................................................ 1139

Consensus CACTGTACTCAAGAATAAATTGAACTCCACCTGAGCTGAAGCACCACCCGAGCCCTCTAG 1200

AY341424.1 ------------------------------------------------------------ 949

c80994_g1_i1 ............................................................ 1199

Consensus GCCACTGGTGAAACTTGAAAAAATCCGCCTGGAACTTCCTTTTAATAAAAA 1251

AY341424.1 --------------------------------------------------- 834

c80994_g1_i1 ................................................... 1250
