# Supplementary material for: Catalogue of stage-specific transcripts in Ixodes ricinus and their potential functions during the tick life-cycle
Source: Parasit Vectors. 2020 Jun 16;13:311. doi: 10.1186/s13071-020-04173-4 (PMC7296661; doi:10.1186/s13071-020-04173-4)
Supplement: Supplementary file 9 — Additional file 9: Alignment S6. Alignment of ixoderin B (GenBank: AY341424.1) query sequence and a truncated corresponding transcript recovered from Ixodes ricinus stage-specific transcriptome assembly (c82323_g13_i1). [file 13071_2020_4173_MOESM9_ESM.docx]

**Additional file 9: Alignment S6.** Alignment of ixoderin B (GenBank: AY341424.1) query sequence and a truncated corresponding transcript recovered from *Ixodes ricinus* stage-specific transcriptome assembly (c82323_g13_i1). Dots indicate agreements, hashes an absence of sequence in the alignment. Underlined sequence in the Consensus represent an ORF.

Additional two sequences (GANP01009384.1,GenBank: EF063563.1) represent ixoderin B5 isoforms found in online nr database with *I. ricinus* transcript used as query. The two sequences show higher similarity with the Ixoderin sequence recovered from our *I. ricinus* assembly than the isoform used as query.

Note the absence of 139 bp at 5’ ORF of the truncated transcript (c82323_g13_i1) recovered from *I. ricinus* stage specific transcriptome assembly.

Consensus ATGTTCGTAGCATTCCTCTTCATACCGGTTCTGGCCGGGRACGTCYTTATGGAAAGCAGT 60

AY643518.3 .......................................G.....C.............. 60

GANP01009384.1 ------------------------------------------------------------

EF063563.1 -......................................A.....T.............. 59

c82323_g13_i1 ------------------------------------------------------------

Consensus TTTCGGCGTGTTCCGGAAATAACCGAAAGACAGTACGGGACGAGRAAAACCTACATGTTA 120

AY643518.3 ............................................G............... 120

GANP01009384.1 ------------------------------------------------------...... 6

EF063563.1 ............................................A............... 119

c82323_g13_i1 ------------------------------------------------------------

Consensus TTTGACCCCTGCAATACGAACAAACCTGGAAATCGAACGRTAAGCTGTTCCCAGATACAA 180

AY643518.3 .....................G.................A.................A.. 180

GANP01009384.1 ......................................G..................... 66

EF063563.1 .......................................A.................... 179

c82323_g13_i1 -------------------....................G.................... 41

Consensus ATGAGGAAACGCAGYCGTA---GCGSTGAATATAMAATTWATCCTCGCAACGATCTGGTA 237

AY643518.3 ......G.......CA.C.CGTTAACAA......A....A.C....TGGG.AC..C.... 240

GANP01009384.1 ..............T.A..---...G........C....T.................... 123

EF063563.1 ..............T....---...G...C....C....A.................... 236

c82323_g13_i1 ..............C.C..---...C........A....T.................... 98

Consensus ACGGTGAGATGTGACATGGATTCCGATGGCGGCGGATGGACAGTKATTCAACGTCGGACC 297

AY643518.3 GAT.CAGC............GA..........T...........G............... 300

GANP01009384.1 ............................................T............... 183

EF063563.1 ............................................G............... 296

c82323_g13_i1 ............................................T............... 158

Consensus GARTATGAAGTCTACGACAACGAATTCGAGAAARACGMWSGAGATTATGAGCKTGGATTT 357

AY643518.3 ..A.......C.C........A.C.........A.G.AAC............G....... 360

GANP01009384.1 ..G..............................G...CTG............T....... 243

EF063563.1 AGA.........A....................A...AACA...........T....... 356

c82323_g13_i1 ..G..............................G..CCTG..........A.GC...... 218

Consensus AAAGYAACCGGGAGCRGTTACTGGATAGGMMWYGAAAATCTTCATGCACTTACGAGTTTT 417

AY643518.3 .C..C.CAA...G.AGC..TG........CAACCC..T........TG..........C. 420

GANP01009384.1 ....T...T......A.............ACTT........................... 303

EF063563.1 ....T..........G.............CAAC......................A...C 416

c82323_g13_i1 ....C......A...A.............ACTT........................... 278

Consensus CCGAACAACCAACAAGCTCTCAGAATCGAACTGACAAGAAAAGGAGCAMCGGAACCGACA 477

AY643518.3 ...G............T.....A.....................---.CA.CG....... 477

GANP01009384.1 ..................................A........A....A........... 363

EF063563.1 ...........G...T..........T...................A.C..A........ 476

c82323_g13_i1 ................................A...............A........... 338

Consensus GTWKTGCTCTATCRCAAATTTMTCGTTGGCTCCAAAAAGGAGRACTACAAGCTGACCATC 537

AY643518.3 ..TG..TA....AA.......CG...C..............AA.G............... 537

GANP01009384.1 ..AT.........G.......A..............GC....G................. 423

EF063563.1 ..TG.......C.G.....C.CAA......C.........G.GG................ 536

c82323_g13_i1 ..AT.........A......CA..............CG....A.T............... 398

Consensus GACGASTACGAGGGTCCTGAMGGTTACGACGCGTTGTCCTACCACAACGGAGAGAAGTTC 597

AY643518.3 .G..GC...C........CCA............C...........-......CCG..... 596

GANP01009384.1 .....G..............C....................................... 483

EF063563.1 .....G..............A.A........................A............ 596

c82323_g13_i1 .C...C............A.C......A................................ 458

Consensus ACCRTCAAGAAGAGCATGACGSARAATCCCGACARAGACAGGTGCTCGGATAGACTAAGT 657

AY643518.3 ...AA..G..GAG--CA....C.A.CA.......AGACA..TGCTC.A............ 654

GANP01009384.1 ...G.................G.G.G........G.....................G... 543

EF063563.1 ...G.................C.G.........GG......C........A......... 656

c82323_g13_i1 ...A.................G.A..........A.....A......CA........... 518

Consensus GGYGGCTGGTGGTTYAAGRAMTGCAACAAGGCAAACCTTAACGGACGTAAATTCRAAYAC 717

AY643518.3 ..C...........C...G.A........................A.......TG..T.. 714

GANP01009384.1 ..T...........T...ACC......C..........................A..C.. 603

EF063563.1 ..T...........T...A.C..............T..................ACCCM. 716

c82323_g13_i1 ..C...........C...G.A.................................G..T.. 578

Consensus GCTTTGGAATTGAAAACATCAAAGTCCCTCGGTATTACCTGGTACATCAAAGACAACGAS 777

AY643518.3 ...........................................................C 774

GANP01009384.1 ....G...............T......................................G 663

EF063563.1 C...C.A.---------.A.....G....G......................G...A..G 767

c82323_g13_i1 ...........................................................C 638

Consensus CAATCCTACTATTACSTYTACGACRGTGTGGAGATGAAGATCAGGGACGACGACTWTGGT 837

AY643518.3 ...............G.T......G..............................T.... 834

GANP01009384.1 ...............C.C......A..............................A.... 723

EF063563.1 G.............TC.T......A................T......T......A.... 827

c82323_g13_i1 ..G............G.C......G..............................T.... 698

Consensus TTCTGCACGGGCKCTTTAAAATCCTAACTTATCTTATTTTTAATCTCTGTTTCGGTGC-- 895

AY643518.3 ..T.........TT..............................T.............-- 892

GANP01009384.1 ............G....T.........--------------------------------- 783

EF063563.1 ............GG...-..G........C.A................C.........AA 886

c82323_g13_i1 ............T..C...G...................C..................-- 756

Consensus ----AAAAAAACACTAAACTGCGCGGAAGTTGCCATTTACAATATGCTCCGTGAATTTCT 951

AY643518.3 ----......G...............................................T. 948

GANP01009384.1 ------------------------------------------------------------ 843

EF063563.1 AAAA..............TC..C.A......ATT......T................... 946

c82323_g13_i1 ----........................................................ 812

Consensus CGC-TTTTTGACTTTTCCCTGCAAACTTTGGAAATAAAATATCACTGSTARAKAAMAMAA 1010

AY643518.3 ...-...........................................G..G.T..C.C.. 1007

GANP01009384.1 ------------------------------------------------------------ 903

EF063563.1 ...T.....T.......T...........------------------------------- 1006

c82323_g13_i1 ...-...........................................C..A.G..A.A.. 871

Consensus TAAAAAAAAAAAAAAAAAAAAAAAAAAAAAA 1041

AY643518.3 ............................... 1038

GANP01009384.1 ------------------------------- 750

EF063563.1 ------------------------------- 975

c82323_g13_i1 ------------------------------- 871
